# Digital and Blended Lifestyle Interventions for Preschool-Aged Children and Families With a Low Socioeconomic Position and the General Population: Scoping Review

**DOI:** 10.2196/86596

**Published:** 2026-06-05

**Authors:** Lea Hohendorf, Tessa Dekkers, Hanneke Kip, Laurence Alpay, Saskia M Kelders

**Affiliations:** 1 Health Psychology & Technology, Faculty of Behavioural, Management and Social Sciences, University of Twente, Drienerlolaan 5, Enschede, Overijssel, 7522 NB, The Netherlands, 49 1708162426; 2 Medical Technology Research Group, Faculty of Healthcare, Sport & Well-being, Inholland University of Applied Sciences, Haarlem, The Netherlands; 3 Department of Research, Stichting Transfore, Deventer, The Netherlands; 4 Optentia Research Focus Area, Vaal Triangle Campus, North-West University, Vanderbijlpark, South Africa

**Keywords:** eHealth, lifestyle interventions, digital health interventions, health behaviors, preschool-aged children, low socioeconomic position, intervention characteristics, effectiveness, intervention evaluation, persuasive systems design

## Abstract

**Background:**

Many unhealthy habits develop in early childhood and can lead to long-term health risks, which disproportionately affect children with low socioeconomic position (SEP). Digital and blended lifestyle interventions can promote healthier lifestyles, yet families with lower SEP remain underrepresented and face unique barriers to healthy behaviors and intervention access. As a result, it remains unclear which intervention characteristics are most effective for these populations.

**Objective:**

This study aimed to identify and map digital and blended lifestyle interventions targeting preschool-aged children and explore how intervention characteristics and reported effectiveness patterns differ between interventions for the general population and low SEP families.

**Methods:**

A search across Scopus, Web of Science, Cochrane Library, ERIC, and ACM Digital Library was conducted. Studies were eligible if they (1) targeted preschool-aged children (6 months to 5 years) or caregivers, (2) evaluated a digital or blended lifestyle intervention, and (3) addressed at least one behavioral domain (nutrition, physical activity, sedentary behavior, sleep, and oral health). Studies focusing on pregnant women, children aged >5 years, or interventions delivered solely nondigitally were excluded. Screening and prioritization were supported by artificial intelligence–assisted software (ASReview; Utrecht University). Data covered intervention targets, populations, theoretical and guideline foundations, delivery modes and settings, and persuasive systems design features. Intervention characteristics and effectiveness categories were synthesized descriptively. The methodological quality of quasi-experimental studies was assessed using the Joanna Briggs Institute critical appraisal tools.

**Results:**

A total of 77 studies describing 54 interventions were included. Of these, 35 targeted the general population, and 19 focused solely on low SEP families. Interventions were similar across groups: typically parent-focused, targeting multiple lifestyle domains, and informed by theories, frameworks, or evidence-based guidelines. Low SEP interventions more often used text messaging, included fewer persuasive design features, and tended toward single delivery channels. Effectiveness findings were mixed; no consistent patterns emerged when interventions were grouped by their characteristics. Among low SEP interventions only, less effective interventions more often targeted multiple behaviors and included more persuasive systems design features.

**Conclusions:**

To our knowledge, this is one of the first reviews to map digital and blended lifestyle interventions for preschool-aged children while comparing intervention characteristics and descriptive effectiveness patterns across general and low SEP populations. The review highlights heterogeneity in intervention characteristics and outcomes and identifies gaps where evidence is limited. These findings underscore the need to better represent low SEP families in intervention research, strengthen understanding of feasibility and contextual fit, and support multilevel approaches that reflect families’ everyday contexts. However, the findings should be interpreted in light of several limitations, including the exclusion of gray literature, partial double-screening, the possibility that artificial intelligence–assisted prioritization may have omitted relevant studies, and variation in the reporting of intervention components across studies.

## Introduction

Unhealthy lifestyle behaviors are already common in preschool-aged children (6 months to 5 years), raising concern as they may shape long-term health trajectories [1,2]. Across regions, only a minority of preschool-aged children, typically fewer than 1 in 10, meet all 24-hour movement guidelines for physical activity, screen time, and sleep [3,4]. Likewise, frequent consumption of sugary snacks and drinks, which contributes to early childhood caries, is already common in this age group [5-7]. These behaviors are linked to an increased risk of overweight, obesity, and other noncommunicable diseases later in life [8-12]. Because routines are established early, intervening in this age group is critical to prevent negative health outcomes and promote healthier lifelong habits.

This need for early prevention is especially urgent among children from families with a low socioeconomic position (SEP), who are disproportionally affected by these health disparities. For example, European studies show that children with less-educated parents are more than twice as likely to be overweight as peers from higher-educated families [13]. SEP, defined by income, education, occupation, and perceived social class [14], strongly shapes health behaviors, as structural barriers such as financial constraints, neighborhood safety, and access to resources can hinder adoption of healthier lifestyles [15,16]. Language barriers and low literacy may also limit access to interventions and their content [17]. Without accounting for families’ contexts, interventions may result in poor engagement and may widen disparities [18]. Despite their greater need, low SEP families remain underrepresented in research and prevention efforts. A systematic overview of existing interventions can help clarify the extent to which this group has been targeted and reveal important gaps in prevention approaches.

More specifically, limited knowledge exists on the characteristics of interventions for preschool-aged children, as well as how such characteristics have been incorporated into their design and delivery. For example, parents are often assumed to be the main target, which makes sense given their central role in shaping children’s behaviors [19]. Yet, questions remain about whether and how other potential stakeholders, such as children themselves or grandparents, have been addressed and what this might mean for intervention relevance and effectiveness [20]. Delivery and design strategies raise similar uncertainties. Traditional formats can create financial and logistical barriers [21,22], while digital approaches offer broader reach and tailoring but face challenges such as low literacy, limited trust, and poor connectivity [18,23-25]. Such barriers can undermine adherence. Blended formats may help address these barriers. Design features such as reminders, feedback, and social support, as described in the persuasive systems design (PSD) framework, may further enhance support [24,26-28]. Yet it remains unclear how these strategies have been implemented in interventions for preschool-aged children or what lessons might be drawn from their application. Taken together, these gaps highlight the need for a systematic overview that consolidates knowledge on intervention characteristics, clarifies which strategies have been attempted, and identifies where important opportunities remain.

To address these gaps, this scoping review provides a comprehensive overview of existing digital and blended lifestyle interventions for preschool-aged children, considering both the general population and families with a lower SEP. low SEP families are of particular interest, as they face greater structural barriers to healthy behaviors but also have the most to gain from effective, well-designed interventions. More insight is needed into what “well-designed” might mean in this context, for example, which intervention characteristics have been most frequently applied, how different target groups have been addressed, and how effectiveness has been reported. An overview may help identify patterns or gaps in these areas, offering useful insights to guide the development of future interventions. Therefore, this review seeks to answer the following research questions: (1) What are the characteristics of existing digital and blended lifestyle interventions aimed at improving lifestyle behaviors in preschool-aged children? (2) What descriptive effectiveness patterns are reported in digital and blended lifestyle interventions targeting preschool-aged children? For both questions, additional investigation was conducted regarding how characteristics and effectiveness levels differ between interventions targeting the general population and those designed for low SEP families.

## Methods

### Scoping Review Methodology

A scoping review was chosen as it is well suited for mapping key concepts, identifying knowledge gaps, and systematically assessing the breadth of existing research in an emerging field [29]. Given that this review aimed to map intervention characteristics and provide a descriptive overview of the effectiveness patterns across a heterogeneous body of studies, a scoping review design was considered most appropriate to provide an overview of the current state of affairs. This review follows the Joanna Briggs Institute (JBI) framework, which expands upon the methodology proposed by Arksey and O’Malley [30] and refined by Levac et al [31]. Additionally, this review adheres to the PRISMA-ScR (Preferred Reporting Items for Systematic Reviews and Meta-Analyses extension for Scoping Reviews) reporting guidelines, and the full checklist is provided in Checklist 1. No protocol was registered for this scoping review.

### Eligibility Criteria

The inclusion and exclusion criteria for this review are provided in Table 1. These criteria were formulated to identify studies examining digital and blended interventions that promote healthy lifestyle behaviors in preschool-aged children (6 months to 5 years). The criteria were developed following the JBI population, concept, and context framework to ensure methodological rigor. Language criteria restricted inclusion to studies published in English. Population criteria focused on interventions targeting preschool-aged children (6 months to 5 years) and/or their caregivers, with additional stakeholders (eg, parents-to-be, educators, and health care professionals) included only when the intervention ultimately targeted improvements in children’s lifestyle behaviors. The concept domain covered two elements: (1) the intervention aim, representing one or more targeted lifestyle behaviors (nutrition, physical activity, sedentary behavior, sleep, and oral health), and (2) the delivery strategy requiring interventions to be delivered digitally or in a blended format. Digital interventions were defined as those including at least one digital component (eg, text messages, app, and website), while blended interventions combined digital and nondigital (eg, in-person sessions and paper-based materials) components. Study type criteria permitted empirical peer-reviewed research reporting effectiveness, development, or implementation of interventions (eg, randomized controlled trials [RCTs], protocols, and feasibility studies). Context criteria were left unspecified to allow inclusion of studies conducted in diverse settings.

**Table 1.** Eligibility criteria used during title, abstract, and full-text screening. The criteria outline the included population (preschool-aged children and/or caregivers), the targeted lifestyle behaviors (nutrition, physical activity, sedentary behavior, sleep, and oral health), the required delivery strategy, and eligible study types.

Aspect	Inclusion	Exclusion
Language	English	Any other language
Population	Target preschool-aged children aged >6 months Target parents of preschool-aged children Target pregnant women and parents-to-be (preventatively) Target any other caretaker of preschool-aged children Family-based Target preschool or kindergarten teachers Target health care professionals Interventions targeting other stakeholders besides children were included only if they indirectly targeted preschool-aged children’s health outcomes	Targets children aged >5 years Targeted pregnant women for the prenatal health of the fetus
Aim	Nutrition Physical activity Sedentary behavior Sleep Oral health	Mental health Management of (chronic) illnesses or disorders (eg, autism and arthritis) Supporting healthier lifestyles in pregnant women Promoting (exclusive) breastfeeding
Delivery strategy	Delivered digitally Delivered in a blended way (digital components combined with in-person or paper-based materials)	Delivered solely in person Delivered solely paper-based
Study type	Peer-reviewed journal papers reporting effectiveness, development, or implementation studies (including RCTs^a^, protocols, pilot studies, and feasibility studies)	Systematic reviews Reports of process evaluations

a RCT: randomized controlled trial.

### Information Sources

A systematic electronic literature search of five databases, namely Scopus, Web of Science, ERIC, ACM Digital Library, and Cochrane Library, was conducted. Each database was searched individually, and no multidatabase vendor platform was used. No study registries (eg, ClinicalTrials.gov), gray literature sources, handsearching, website browsing, or other supplementary search methods were used. Backward citation searching of included studies was performed to identify additional potentially relevant records. No study authors or experts were contacted to identify additional studies or obtain additional data. The original searches were executed in November 2023, with updated searches using the same strategy and databases conducted in May 2025 and January 2026 to ensure inclusion of recently published studies. No date limits were applied to the initial search, and the update ranges were restricted to periods not previously covered.

### Search Strategy

The search strategy and its reporting followed the PRISMA-S (Preferred Reporting Items for Systematic Reviews and Meta-Analyses literature search extension) guidelines [32]; the complete PRISMA-S checklist is provided in Checklist 2. An information specialist from the University of Twente was consulted for the construction of the search strategy and the selection of databases. No search strategies from prior reviews were reused or adapted. The search strategy included keywords related to the concepts of digital and blended interventions, lifestyle behaviors, lifestyle interventions, and the target group (eg, preschool children and parents). No methodological search filters were applied. Search results were imported into Mendeley (Elsevier; initial search and the May 2025 update) and Zotero (Corporation for Digital Scholarship; January 2026 update), where duplicates were identified, manually verified, and removed. The complete search strategy used for all databases is available in Multimedia Appendix 1.

### Selection of Sources of Evidence

The search results from all databases were combined and exported into the reference manager Mendeley, where duplicate records were removed. The final set of search results was then imported into ASReview (version 1.2.1; Utrecht University) for the selection of eligible studies. ASReview is an artificial intelligence (AI)-assisted tool designed to aid researchers in systematically reviewing literature [33]. To train the AI model, it was provided with prior knowledge in the form of 14 preselected papers (refer to Multimedia Appendix 2), labeled as relevant or irrelevant by the investigating researcher. ASReview uses an active learning algorithm that continuously reprioritizes records to present those most likely to be relevant first. As the model stabilizes, the probability of identifying additional relevant papers becomes very low. To balance comprehensiveness and efficiency, we used a mixed stopping rule: screening was terminated after a minimum of 10% of the dataset had been reviewed, and 150 consecutive records were irrelevant. This pragmatic rule follows a similar rationale to those used in other AI-assisted screening procedures (eg, SAFE heuristic) [34,35].

The updated search was conducted using the same search strategy and databases, and newly identified records were screened using the same procedure. The selection process consisted of a two-step screening process, including (1) title and abstract screening and (2) full-text screening. During the title and abstract screening, 2 authors (LH and TD) reviewed 200 papers and made inclusion and exclusion decisions on 187 of these. Agreement was observed for 97% of overlapping records (κ=0.90), indicating almost perfect agreement [36]. Because the first 100 abstracts were reviewed jointly before independent screening began, Cohen kappa should be interpreted as an upper-bound estimate of interrater reliability. Disagreements or uncertainties were discussed until consensus was reached; if no agreement could be found, a third author (HK) was consulted. The remaining abstracts were then screened by the first author (LH) until a total of 928 abstracts had been reviewed, and 150 consecutive papers were deemed irrelevant. In the full-text screening phase, a random selection of 12 full-text papers (10% of all papers) was independently reviewed against the inclusion and exclusion criteria by 2 authors (LH and TD), with any doubts or disagreements discussed and resolved. Cohen κ was 0.71, indicating substantial agreement between reviewers [35]. If consensus could not be reached, the third author (HK) was consulted. Finally, data extraction from the remaining eligible full-text papers was conducted by the first author (LH).

### Data Charting Process

A data extraction form was developed to systematically collect key information relevant to the review questions (refer to Table 2). The first author (LH) conducted the data charting, with uncertainties or inconsistencies discussed and resolved with the second author (TD). Initially, data were charted deductively using predefined data items.

**Table 2.** Data items coded for data extraction. The table summarizes the population, study, and intervention characteristics extracted from each included paper, with accompanying explanations detailing how each item was defined and operationalized for this review.

Data item	Explanation
Population	Coded as “low SEP^a^” intervention only if the studies explicitly stated an aim to reach vulnerable populations; otherwise, they were coded as “general population.”
Study design	For example, a randomized controlled trial or protocol.
Study research objectives	Research aims or objectives.
Study outcomes	Primary outcomes.
Intervention name	Name of intervention.
Intervention objectives	The intervention objectives of each intervention were recorded.
Target behaviors	Moreover, the specific target behaviors (refer to Table 1) were documented under their behavior categories.
Target group	For example, “parents,” “caregivers,” or “family.”
Theories, frameworks, and evidence-based guidelines	For example, behavioral frameworks and theories, behavior change techniques, or evidence-based guidelines.
Strategy and channel	Delivery strategy refers to interventions being delivered either fully digitally or in a blended way. Blended refers to a combination of digital components and in-person or paper-based aspects. Concerning the delivery channel, we documented the usage of any tool to deliver the intervention content, such as a “smartphone app” or “website.”
Delivery environments	For example, “daily life” or “in a primary care clinic.”
Persuasive features	The persuasive features implemented in the interventions were documented and coded according to the persuasive systems design framework by Oinas-Kukkonen and Harjumaa [28]. Identified features were coded into four categories, namely, primary task support, dialogue support, credibility support, and social support.

a SEP: socioeconomic position.

### Data Items

The predefined data items included target group, persuasive features, and theoretical and conceptual basis. A complete overview of all extracted variables is provided in Table 2.

### Critical Appraisal of Individual Sources of Evidence

The methodological quality of studies was assessed using the JBI critical appraisal tools for RCTs and quasi-experimental studies. These checklists evaluate the extent to which a study minimizes bias in its design, conduct, and analysis. The RCT checklist contained 13 questions, while the quasi-experimental checklist had 9 questions. To ensure reliability, 10% of studies were independently screened by both the first and second authors, with discrepancies resolved through discussion. Papers were classified based on the approach used by Helmyati et al [37]: (1) low risk of bias (RCTs with 10‐13 “yes” answers or quasi-experimental studies with 7‐9), (2) medium risk of bias (RCTs with 7‐9 “yes” answers or quasi-experimental studies with 5‐6), and (3) high risk of bias (RCTs with 0‐6 “yes” answers or quasi-experimental studies with 0‐4). Critical appraisal was conducted to describe study quality and was not used as a basis for excluding studies.

### Synthesis of Results

The extracted information was coded inductively to identify and group similar characteristics across interventions. To avoid overrepresenting interventions with multiple components, each characteristic was recorded only once per intervention, even if it was implemented in several ways (eg, personalization delivered via both email and app was coded as a single instance). To ensure consistent and reproducible categorization of target populations, interventions were classified as targeting low SEP families only when this was explicitly stated by the study authors. This criterion was chosen to minimize subjective inference and enable mutually exclusive grouping across studies. The synthesis was descriptive in nature. To address the second study objective, interventions evaluated using a quasi-experimental design were classified as more effective, less effective, or ineffective based on the criteria established by Morrison et al [38]. Using this framework, interventions were categorized as more effective if they led to improvements in most outcome measures and/or were at least as effective as comparison groups and more effective than no-intervention groups. Interventions were considered less effective if they led to improvements in only a minority of outcomes or were less effective than comparison groups but still outperformed waiting-list groups. Finally, interventions were classified as ineffective if they resulted in no measurable improvements or change. This categorization was conducted based on the outcome patterns reported in the included papers. No statistical reanalysis was performed, and the categorization is intended to provide an overview of reported patterns rather than to support causal conclusions or claims of statistical predominance.

### PSD Features

Persuasive elements within the intervention design were analyzed using the PSD framework [28] to assess how digital and blended interventions encourage behavior change. The framework classifies persuasive features into four principles: primary task support, dialogue support, system credibility, and social support. PSD features were coded deductively using the framework. Even when authors did not explicitly identify intervention features as persuasive, they were documented if present based on the description of the interventions and available screenshots. A full list of PSD features and their respective categories is available in Multimedia Appendix 3.

### Ethical Considerations

Ethical approval was not required for this scoping review because the study analyzed publicly available published literature and did not involve human participants or identifiable personal data.

## Results

### Selection of Sources of Evidence

**Figure 1.**
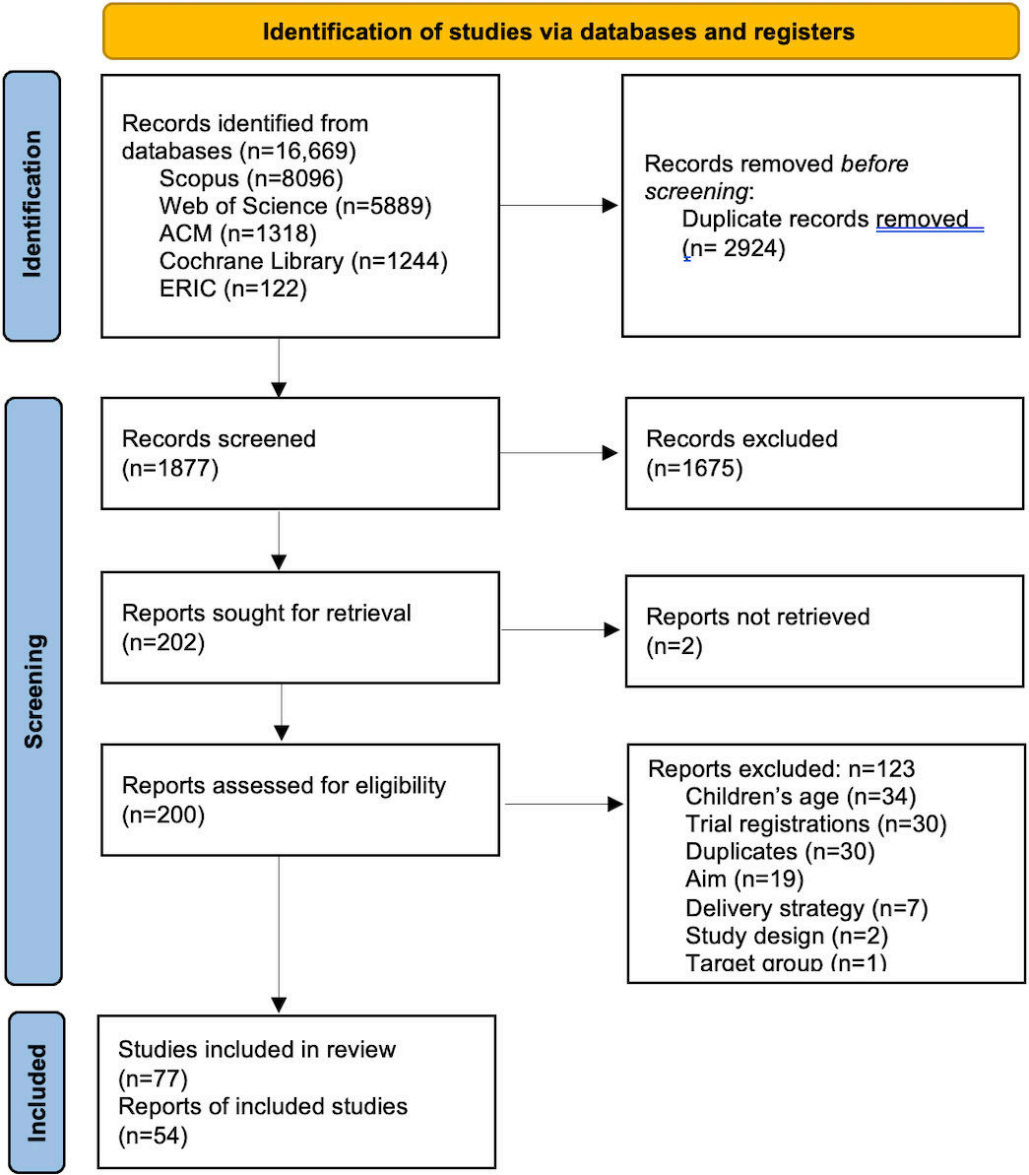
PRISMA (Preferred Reporting Items for Systematic Review and Meta-Analyses) flow diagram presenting identification and selection of papers for the scoping review of digital and blended lifestyle interventions for preschool-aged children. The search process consisted of an initial search (November 2023), an updated search (May 2025), and a second updated search (January 2026); numbers are combined for clarity.

The full search strategy, number of included papers, and reasons for exclusion are provided in Figure 1. The initial and updated search together yielded more than 13,000 records, from which 77 papers describing 54 interventions were included. The most common reasons for exclusion were targeting children outside the 6-month to 5-year age range, trial registrations, duplicate records, and a focus on breastfeeding or parental health (physical or mental).

### Characteristics of Sources of Evidence

An overview of the 77 included studies is provided in Multimedia Appendix 4, while Table 3 provides a summary of study and intervention characteristics across population groups. Of these, 34 (44%) were protocols for experimental studies, 35 (46%) were pre- and quasi-experimental studies, and 8 (10%) were feasibility, acceptability, and usability studies. The protocols primarily described intervention development and outlined the design and methodology of planned effectiveness trials. Among the 35 pre- and quasi-experimental studies, most used an RCT design (n=22, 63%), followed by cluster RCTs (n=7, 20%). These studies mainly evaluated intervention effects on targeted lifestyle behaviors and anthropometric outcomes, such as BMI.

**Table 3.** Overview of study and intervention characteristics across population groups (general population and low socioeconomic position [SEP]), including study design; target behaviors; target group; theories, frameworks, and evidence-based guidelines; strategy; channel; delivery environment; and persuasive features. Values are reported as numbers (n) and percentages (%). Counts represent how often a characteristic was reported or present across the included studies and interventions.

Intervention characteristic	General population, n (%)	Low SEP^a^, n (%)	Total, n
Study design			
Protocols	25 (74)	9 (26)	34
Pre- and quasi-experimental studies	24 (67)	12 (33)	36
Feasibility, acceptability, and usability studies	4 (57)	3 (43)	7
Target behaviors			
Nutrition	11 (61)	7 (39)	18
Physical activity	1 (100)	0 (0)	1
Sedentary behavior	1 (100)	0 (0)	1
Sleep	3 (100)	0 (0)	3
Oral health	1 (100)	0 (0)	1
Combination	18 (60)	12 (40)	30
Target group			
Parents or caregivers	28 (67)	14 (33)	42
Others	7 (58)	5 (42)	12
Theories, frameworks, and evidence-based guidelines			
Evidence-based recommendations, guidelines, and previous research	16 (70)	7 (30)	23
Social cognitive theory	14 (78)	4 (22)	18
BCTs^b^	15 (83)	3 (17)	18
Nothing reported	3 (50)	3 (50)	6
Others	37 (70)	16 (30)	53
Strategy			
Digital	18 (64)	10 (36)	28
Blended	17 (65)	9 (35)	26
Channel			
Application-based components	12 (71)	5 (29)	17
Browser-based components	13 (72)	5 (28)	18
Human-delivered components	14 (61)	9 (39)	23
Voice or text messages	14 (58)	10 (42)	24
Emails or e-newsletters	2 (67)	1 (33)	3
Social media	5 (42)	7 (58)	12
Materials	6 (60)	4 (40)	10
Delivery environment			
Daily life	24 (67)	12 (33)	36
Others	11 (61)	7 (39)	18
PSD^c^ features			
Primary task support	36 (78)	10 (22)	46
Dialogue support	31 (74)	11 (26)	42
System credibility support	8 (80)	2 (20)	10
Social support	9 (53)	8 (47)	17

a SEP: socioeconomic position.

b BCT: behavior change technique.

c PSD: persuasive systems design.

### Synthesis of Results

#### Overview of Intervention Characteristics

Overall, 54 interventions were included in the scoping review. Their key characteristics are summarized in Multimedia Appendix 5. The majority targeted families in the general population (n=35, 65%), while 19 (35%) interventions focused solely on low SEP families. A typical intervention, regardless of the target group, addressed multiple behavior categories, was parent-focused, informed by theories or evidence-based guidelines, delivered digitally, and integrated into daily-life contexts. For instance, Wu et al [39] (general population) and Lee et al [40] (low SEP) targeted nutrition and physical activity through digital channels such as social media apps, text messages, and websites to engage parents and caregivers in their daily routines. Both interventions were based on evidence-based recommendations, guidelines, and previous research or social cognitive theory. However, a few interventions differed from this general pattern. For example, the Barns matmot (version 2.0) intervention targeted only nutrition-related behaviors and involved kindergarten staff alongside caregivers, combining web components with in-person delivery, such as providing warm meals [41].

**Figure 2.**
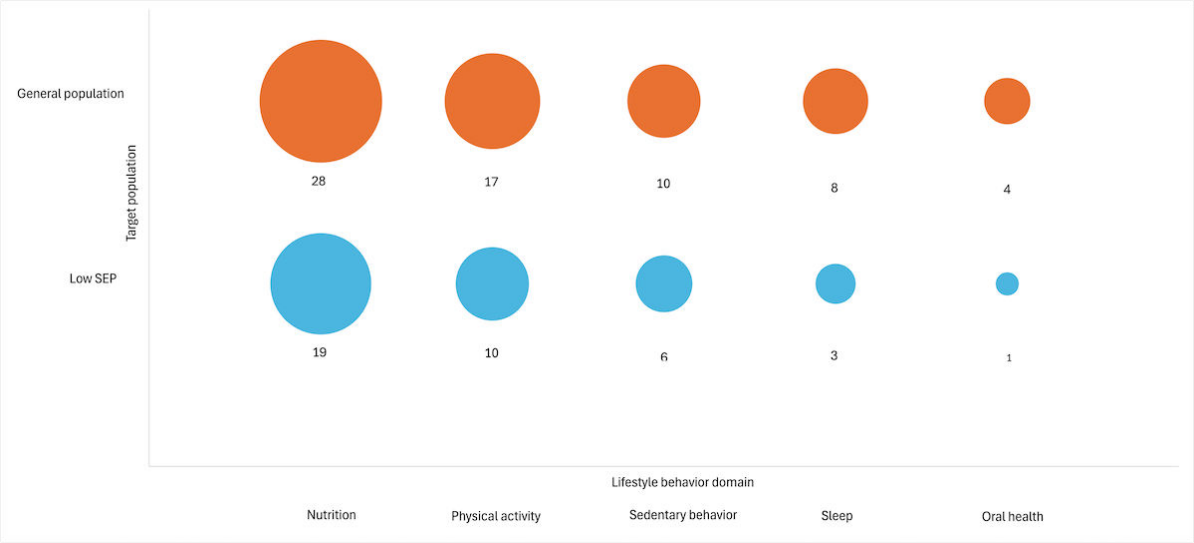
Evidence gap map showing the distribution of targeted lifestyle behavior domains in digital and blended lifestyle interventions for preschool-aged children across population groups (general population and low socioeconomic position [SEP] families).

**Figure 3.**
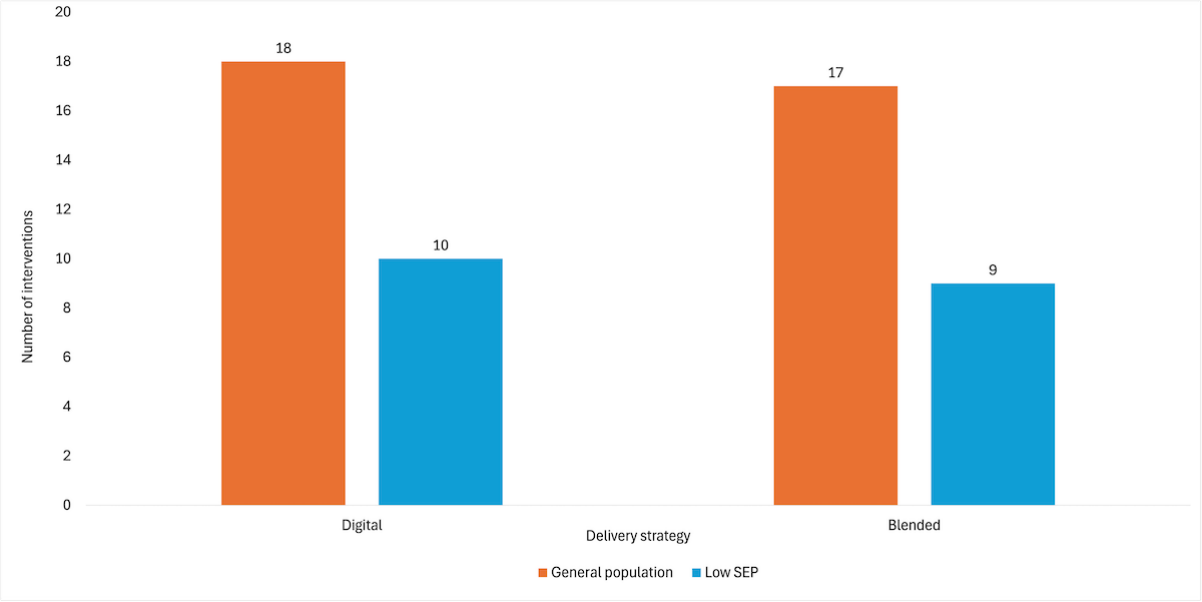
Distribution of included interventions by delivery mode (digital vs blended) stratified by population group (general population vs low socioeconomic position [SEP]).

A gap map was created to support the descriptive synthesis of intervention characteristics across the general population and low SEP interventions (refer to Figure 2). Nutrition was the most frequently targeted domain in both groups. Physical activity and sedentary behavior were also common targets, whereas sleep and oral health were addressed less frequently, particularly in low SEP interventions. The complementary bar chart in Figure 3 illustrates the distribution of digital vs blended delivery strategies across the 2 population groups. Overall, digital interventions were slightly more common than blended ones, and the distribution was nearly equivalent within each population group.

#### Target Behaviors

Many interventions in both groups targeted multiple behavior categories, ranging from 2 to 4 (general population: 18/35, 51% and low SEP: 12/19, 63%). Illustrative examples of how these target behaviors were presented within interventions are provided in Multimedia Appendix 6. For example, MINISTOP (version 2.0) [42] addressed nutrition, physical activity, sedentary behavior, sleep, and oral health in the general population, while Futuros Fuertes [43] focused on nutrition, sedentary behavior, and sleep in low SEP families. A total of 17 (49%) interventions in the general population group and 7 (37%) interventions in the low SEP group focused on a single behavior. Within single-behavior interventions, nutrition-related behaviors were the most common focus (general population: 11/17, 65% and low SEP: 7/7, 100%), with objectives such as reducing sugar-sweetened beverage intake, limiting unhealthy snacking, promoting fruit and vegetable consumption, and supporting responsive feeding practices. Only a few general population interventions exclusively addressed other behaviors, including sleep (n=3, 17%), oral health (n=1, 6%), physical activity (n=1, 6%), and sedentary behavior (n=1, 6%).

#### Target Group

In both groups, interventions primarily targeted parents as agents of behavior change for their children (general population: 28/35, 79% and low SEP: 15/19, 79%). A smaller number of interventions also involved other caregivers or intermediaries. These included health care professionals (general population: 1/35, 3%), early childhood educators (general population: 2/35, 6%), preschool staff together with parents or children (general population: 3/35, 9% and low SEP: 1/19, 5%), and multiple caregivers such as parents and grandparents (general population: 1/35, 3% and low SEP: 3/19, 16%). For example, the general population interventions Barns matmot 2.0 [41] and Go NAPSACC [44] engaged preschool staff alongside parents and children, while FUNS [45] targeted mothers, fathers, and grandparents in low SEP families.

#### Theories, Frameworks, and Evidence-Based Guidelines

The majority of interventions reported using theories, frameworks, and guidelines to inform their content and delivery (general population: 32/35, 91%, and low SEP: 16/19, 84%), with many incorporating multiple (general population: 22/32, 69%, and low SEP: 9/16, 56%). Among the reported approaches, evidence-based guidelines and previous research were the most frequently documented, forming the basis for intervention content and rationale in nearly half of all studies (general population: 16/32, 50%, and low SEP: 7/16, 44%). For instance, both MINISTOP (version 2.0; general population) and the Healthy Future Program (low SEP) relied on evidence-based guidelines from organizations such as the World Health Organization (WHO) and the American Academy of Pediatrics [42,46]. Social cognitive theory was the second most frequently cited framework (general population: 14/32, 44%, and low SEP: 4/16, 25%). The Greenlight Plus intervention [47] (general population), for example, combined social cognitive theory, active learning, and empirical evidence and guidelines, alongside behavior change techniques (BCTs) such as goal-setting and self-monitoring. Meanwhile, the GROW intervention [48] (low SEP) drew on the Centers for Disease Control and Prevention’s theory and social cognitive theory and incorporated BCTs such as self-monitoring, problem solving, and goal-setting.

In addition to these broader frameworks, a subset of interventions reported using BCTs, drawing on frameworks such as the Coventry, Aberdeen, and London-Refined taxonomy [49], the behavior change wheel [50], or the BCTs taxonomy v1 [51] (general population n=13/32, 41%, and low SEP n=3/16, 19%). A total of 9 interventions (general population n=6/13, 46%, and low SEP n=3/3, 100%) reported on the BCTs they used. The most frequently used BCTs were goal-setting (general population n=4/6, 67%, and low SEP n=2/3, 67%), self-monitoring (general population n=3/6, 50%, and low SEP n=2/3, 67%), and feedback (general population n=3/6, 50%, and low SEP n=1/3, 33%). For example, Go NAPSACC (general population) included both self-monitoring and feedback techniques [44] (refer to Multimedia Appendix 6).

### Delivery Strategy and Channel

Slightly more than half of the interventions in both groups used a digital-only strategy (general population: 18/35, 51%, and low SEP: 10/19, 53%), while the other half adopted blended approaches, combining digital and nondigital components. General population interventions predominantly used multiple delivery channels (21/35, 60%), including websites, apps, online modules, and text messages, whereas low SEP interventions were almost evenly split between single (9/19, 47%) and multiple channels (10/19, 53%). In the low SEP group, commonly used digital tools included voice or text messages (10/19, 53%) and social media platforms (7/19, 37%) such as Facebook (Meta Platforms, Inc) groups and forums. For example, Food4Toddlers (general population) delivered content through a web-based platform and mobile app, providing parents with interactive modules, videos, recipes, and a discussion forum to support healthy food and eating environments in their daily routines [52]. Meanwhile, IIMAANJE (low SEP) or TEXT2COPE (general population; refer to Multimedia Appendix 6) used weekly voice and text messages to engage both mothers and fathers, offering practical guidance on infant and young child feeding practices that could easily be integrated into daily caregiving [53,54].

Among interventions using blended approaches, in-person components, such as counseling sessions, educational workshops with professionals or researchers, or delivery of content to children in classes, were common (general population: 14/35, 49%, and low SEP: 8/19, 47%). Other blended approaches provided additional physical materials, including pamphlets, measuring cups, water bottles, or magnets, to reinforce digital content and support behavior change in daily routines. The Ready, Set, Gulp! intervention by Lewis et al [55], for example, combined a smartphone app with a water promotion toolkit, including water bottles, stickers, and a booklet (refer to Multimedia Appendix 6).

### Delivery Environment

Most interventions were delivered in a single environment, primarily daily life, for both the general population (24/35, 69%) and low SEP groups (12/19, 63%). For example, Early Food for Future Health [56] sent parents monthly emails linking to a secure website with videos and recipes designed for flexible, at-home viewing, while Samen Happie! [57] provided parents with an app containing age-based modules and interactive challenges to seamlessly integrate into daily routines. However, some interventions also incorporated additional delivery settings, including primary care clinics, daycare centers or preschools, community or organizational centers, or other unspecified locations for in-person sessions (refer to Multimedia Appendix 5).

### Persuasive Features

None of the included studies explicitly reported using PSD features. However, 45 interventions (general population: 30/35, 86%, and low SEP: 15/19, 79%) provided sufficiently detailed descriptions to enable deductive coding based on the PSD framework [28] (refer to Multimedia Appendix 5). The number of PSD features implemented ranged from 1 to 7 in general population interventions (mode=3) and 1 to 6 in low SEP interventions, where a single feature was most common (mode=1). Multiple PSD features were more frequent in general population interventions (22/30, 73%), whereas low SEP interventions were almost evenly split between using one and multiple features (one: 7/15, 47%, and multiple: 8/15, 53%). For example, MINISTOP (version 2.0; general population) combined reminders, reduction, and rewards, while Cooking Matters (low SEP) relied solely on social learning to promote healthy behaviors [42,58]. Illustrative screenshots demonstrating how these PSD features were implemented are provided in Multimedia Appendix 6. Across both groups, the most commonly incorporated features were suggestions and reminders, which are both part of the dialogue support category (general population: 14/30, 47%, and low SEP: 5/15, 33%). For example, the Nenne Navi (general population) included suggestions by providing advice to the parent [59], while the TEXT2COPE (general population) intervention included reminders as part of its text messages [53]. Notably, low SEP interventions also frequently included social facilitation (8/15, 53%), which was exclusively implemented through social media components.

### Effectiveness Categorization and Intervention Characteristics

Overall, 36 quasi-experimental studies were assessed for methodological quality using the JBI appraisal tools. The full appraisal details are provided in MultimediaAppendices 7  8. Out of these, 16 studies had a low risk of bias, 18 had a medium risk, and 2 studies had a high risk. The most frequent sources of bias were self-reported outcomes, lack of blinding (often not applicable to digital interventions), and absence of intention-to-treat analyses. No clear pattern was observed between study quality and effectiveness classifications: both low- and medium-risk studies included a mix of more effective, less effective, and ineffective interventions (low risk: more effective 7/16, 44%, less effective 7/16, 44%, and ineffective 2/16, 12%; medium risk: more effective 8/18, 44%, less effective 7/18, 39%, and ineffective 3/18, 17%). The 2 high-risk studies were classified as more effective and less effective. Importantly, methodological quality appraisal and effectiveness categorization represent distinct assessments: trial rigor was evaluated using JBI tools, whereas effectiveness reflects only the outcome patterns reported by the original studies.

A total of 36 interventions were assessed for their effectiveness in quasi-experimental trials, alongside 2 follow-up studies evaluating sustainability. As noted in the Methods section, this effectiveness categorization reflects descriptive outcome patterns reported in the original studies. No statistical reanalysis was performed, and the categorizations should not be interpreted as inferential evidence or claims of causal predominance. An overview of the effectiveness categories per target population is provided in Table 4. Overall, interventions targeting the general population were more often categorized as more effective (13/24, 54%) compared to interventions targeting low SEP families (3/12, 25%). Meanwhile, a larger proportion of low SEP interventions were categorized as less effective (9/12, 75%) compared to general population interventions (6/24, 25%). Of the 2 follow-up studies assessing longer-term outcomes, neither MINISTOP [60] nor Early Food for Future Health [61] maintained effects at 12-month follow-up and were therefore coded as ineffective. No single study reported both short- and long-term effects within the same publication; long-term outcomes were only available through separate follow-up studies and were therefore classified independently.

**Table 4.** Effectiveness categorizations of included interventions stratified by target population (general population vs low socioeconomic position [SEP]), reported as number and percentage of interventions in each category.

Effectiveness categorization	General population, n (%)	Low SEP,^a^ n (%)	Total, n (%)
More effective	12 (60)	3 (25)	15 (47)
Less effective	5 (25)	9 (75)	14 (44)
Ineffective	3 (15)	0 (0)	3 (9)
Total	20 (63)	12 (37)	32 (100)

a SEP: socioeconomic position.

When comparing intervention characteristics across the 3 effectiveness categories (more effective, less effective, and ineffective), few consistent descriptive patterns were observed. However, some differences were seen, particularly in interventions targeting low SEP families. For instance, all 3 more effective low SEP interventions (100%) each incorporated only a single PSD feature, with 2 relying exclusively on social learning. In contrast, less effective low SEP interventions were more frequently characterized by multiple PSD features (5/8, 63%). Among these, suggestions and reminders were the most common (3/8, 38%). For example, FirstStep2Health [62] used social learning through a Facebook-delivered program, virtual meetings, and text messages to engage parents and caregivers, while the intervention by Lee et al [40], which combined suggestions, reminders, and self-monitoring, was categorized as less effective. These observations represent an exploratory descriptive pattern and should not be interpreted as causal or prescriptive design conclusions.

Target behaviors appeared to vary descriptively across effectiveness categories. Less effective low SEP interventions more frequently targeted multiple behavior categories (6/9, 67%), whereas interventions in other effectiveness categories, including more effective low SEP interventions, more effective general population interventions, less effective general population interventions, and ineffective general population interventions, were more evenly split between single and multiple target behaviors. For example, Samen Happie! [57], Healthy Children, Strong Families 2 [63], and Futuros Fuertes [43] targeted multiple behaviors such as nutrition, sleep, sedentary behavior, and physical activity. Next, more effective low SEP interventions used blended approaches slightly more often (2/3, 67%). Less effective interventions were predominantly digital-only (6/9, 67%), often relying on voice or text messages (4/6, 67%). For example, communities for healthy living [64] used a blended approach combining education sessions, various materials, online components, and a Facebook group to support parents and caregivers in obesity prevention. In contrast, Cooking Matters [58], Downs et al [65], and NUTRES [66] relied solely on digital channels such as text, voice messages, and social media.

### Feasibility, Acceptability, and Usability

Overall, 13 studies assessed feasibility, acceptability, or usability outcomes, including 8 targeting the general population (MINISTOP [version 2.0] [67], TEXT2COPE [53], Nenne Navi [68], Mini Movers [69], Lewis et al [55], He et al [70], Ferdous et al [71], and Ihab et al [72]) and 5 focusing on families from low socioeconomic backgrounds (FUNS [45], Samen Happie! [73], Downs et al [65], Happy Family, Healthy Kids [74], and Ling et al [75]). Regarding delivery, 5 interventions were fully digital and 8 used blended strategies that combined digital components with in-person or paper-based elements (refer to Table 3). Generally, both digital and blended programs were well received, though blended formats were more frequently evaluated and tended to yield more detailed insights.

Across studies, participants emphasized several positive aspects. Digital interventions were praised for their accessibility, convenience, and ease of integration into daily routines. MINISTOP (version 2.0) [67] and Nenne Navi [68] demonstrated high usability, cultural reach, and user satisfaction, while FUNS [45] showed strong preimplementation acceptability and cultural relevance among low SEP parents. Blended programs such as TEXT2COPE [53], Mini Movers [69], and Lewis et al [55] were valued for structured goal-setting, reminders, and opportunities for interaction and feedback. Interventions for low SEP families, including Happy Family, Healthy Kids [74] and Downs et al [65], achieved high acceptability when materials were culturally tailored and presented through accessible or low-literacy-friendly formats.

Challenges differed by population and context. For general population interventions, issues most often related to time or scheduling demands associated with interactive or in-person components [69-71] and the need for sustained motivation after initial enthusiasm [67,68]. Technical constraints, such as device and connectivity, occasionally affected blended delivery [71]. Among low SEP families, barriers were more structural, including limited phone ownership, unstable network access, and competing work or caregiving demands [65,74]. Engagement sometimes declined after initial uptake, as seen in Samen Happie! [73], despite moderate acceptability scores.

Most studies assessed feasibility and acceptability after participants had used the intervention within a pilot or trial context, focusing on user experience rather than real-world uptake. Only FUNS [45] and Ihab et al [72] examined these aspects formatively to inform later optimization. Additionally, Ling et al [75] reported that children continued using the mindfulness and movement activities after the intervention period, indicating sustained engagement with the child-directed components. Overall, the evidence suggests that interventions for families with young children are feasible and acceptable across populations, although positive evaluations during trials do not necessarily predict sustained use once research involvement or structured support ends.

## Discussion

### Summary of Evidence

This scoping review identified and compared the characteristics of digital and blended lifestyle interventions for preschool-aged children from 6 months to 5 years. Additionally, it examined differences between interventions for the general population and those targeting low SEP families. Across 54 interventions, characteristics were largely similar across populations and effectiveness categories, though more programs targeted the general population. A typical intervention addressed multiple behaviors (eg, nutrition, physical activity, and sleep) and centered on parents as primary agents of change. Most drew on evidence-based guidelines, such as the WHO recommendations on physical activity, sedentary behavior, and sleep for children aged <5 years [8], and theories such as social cognitive theory. Delivery mainly relied on digital channels, including websites, apps, and text messages. Overall, only minor descriptive tendencies were observed. General population interventions tended to include more persuasive features, whereas more effective low SEP interventions used fewer PSD features, and less effective low SEP interventions more often targeted multiple behavior categories. Since no consistent patterns emerged, observed effectiveness categories may relate to additional factors, such as how well interventions align with families’ everyday contexts, routines, and resources. Although most interventions were considered feasible and acceptable in trial settings, these findings may not fully reflect their usability or sustainability in real-world conditions. Understanding these contextual influences, particularly in low SEP settings, might be essential for creating usable and sustainable interventions in practice. While contextual fit was not directly assessed in this review, health-behavior frameworks emphasize that families’ engagement with interventions is shaped by everyday contextual conditions such as routines, competing demands, and environmental or structural constraints [50,76]. From this perspective, contextual fit offers a useful lens for interpreting the heterogeneous descriptive patterns observed in this review. However, this should be viewed as a theory-informed interpretation rather than an empirically tested determinant. Therefore, more research in which contextual fit is used as a determinant is necessary.

This review found that most digital and blended interventions for preschool-aged children target the general population, with limited focus on socioeconomically disadvantaged families. This imbalance echoes earlier observations: despite recognition of health inequalities, disadvantaged groups appear to remain underrepresented in digital health research [77]. It may seem counterintuitive, given that they face higher rates of preventable health issues and could particularly benefit from intervention efforts [13,78]. Limited focus may reflect persistent challenges such as recruitment and retention difficulties, funding priorities, and assumptions about digital access [25]. Yet, greater representation on its own may not be sufficient. Interventions developed without attention to families’ everyday contexts risk offering limited benefit or even unintentionally reinforcing inequalities [79,80]. Our review also suggests that feasibility, acceptability, and usability studies have so far focused mainly on general population interventions. Only a small number have paid attention to these aspects in low SEP interventions, leaving little insight into how low SEP families might perceive and engage with such tools. Future research could therefore place more emphasis not only on improving recruitment, but also on systematically examining these families’ experiences. Involving them early in development may help ensure that interventions are more likely to reflect lived realities and contribute to equitable outcomes. Co-design methods that actively engage these families early in the development process offer a promising avenue for incorporating their experiences and preferences and ensuring that interventions are contextually relevant.

Building on this, the review also considered intervention effectiveness. Interventions developed specifically for low SEP families were more often classified as less effective than those for the general population, though there were exceptions. Notably, some interventions originally designed for the general population appeared more effective in disadvantaged groups once adapted to families’ preferences and contexts. For example, the MINISTOP intervention was initially rated as less effective [81]. A revised version (MINISTOP version 2.0), adapted and translated to be more culturally sensitive and accessible, was more effective in a multiethnic, socioeconomically diverse sample [42]. This may indicate that adapting existing programs can sometimes be a more efficient route than creating entirely new ones. At the same time, interventions focusing solely on individual behavior change, which was the case for most interventions in this review, may be overly optimistic, since families’ choices are shaped by broader social and environmental circumstances. The social ecological model highlights how factors at multiple levels—individual, family, community, and policy—interact to influence health [76,82]. From this perspective, individual-level strategies alone may be insufficient in disadvantaged contexts, as suggested by prior research [83,84]. While some low SEP interventions in this review were categorized as less effective, these observations are descriptive and should not be interpreted as causal evidence. Many low SEP individuals describe their lifestyles as “logical” responses to structural constraints [15], underscoring the need to address surrounding conditions alongside individual choices. Future efforts could therefore focus on adapting the content and design to support individual behavior change more effectively by fitting families’ contexts. Additionally, more attention could be paid to developing strategies at the community and policy levels to create conditions for sustainable impact. More specifically, more evidence is needed on how community- and policy-level strategies can complement and be combined with family-based interventions to create more supportive environments for sustainable behavior change.

Most interventions in this review focused on parents or caregivers as agents of change, consistent with social cognitive theory, which emphasizes observational learning, modeling, and the reciprocal influence between parents’ and children’s behaviors. This approach is supported by evidence of parental influence on child health behaviors [85-87]. While parents play a crucial role, involving children more directly may help them internalize positive behaviors and build lasting habits. Developmental frameworks emphasize children’s active participation in routines as critical for independence and self-regulation [88-90]. Digital tools offer ways to support such engagement. Applying principles from design thinking can make these tools more effective by grounding them in empathy, co-design, and iterative testing with children. Such approaches can lead to more engaging and developmentally appropriate experiences that may also foster a sense of ownership and sustained motivation [91-93]. Playful, interactive formats such as gamified apps have shown promise in early childhood settings. For example, Mole’s Veggie Adventures engaged preschoolers and promoted healthier eating [94]. While these approaches may show promise, sustaining behavior change remains a well-recognized challenge [95,96], as also reflected in this review. Looking beyond the home to settings such as preschools or daycare centers can offer additional opportunities to embed interventions [97-99]. Some of the included studies in this review have already adopted blended formats, including preschools or clinic settings, with mixed results, suggesting that effectiveness may be influenced by implementation quality and contextual fit. Aligning components across home, care, and community settings may help reinforce habits over time [100]. Future research should therefore examine how collaboration among several stakeholders, such as parents, children, educators, health care professionals, and community leaders, can best support intervention sustainability and lasting impact.

### Strengths and Limitations

A key strength of this review is its tailored search strategy. Because evidence on digital and blended lifestyle interventions for preschool-aged children is spread across multiple disciplines, we used a multidisciplinary search strategy and searched databases spanning public health, psychology, medicine, education, and technology (Scopus, Web of Science, Cochrane, ERIC, and ACM Digital Library). The search strategy was carefully developed with all authors and an information specialist and refined collaboratively, increasing the likelihood that relevant studies were captured from fragmented literature.

Several limitations should be noted. First, restricting the search to peer-reviewed literature may have introduced bias by excluding gray literature, such as program evaluations and organizational reports, which often provide valuable but inconsistently reported insights. Second, only a subset of records (200 papers during title and abstract screening and approximately 10% of full-text papers) were double-screened, though inclusion and exclusion decisions were discussed in detail to improve consistency. The use of AI-assisted screening (ASReview) may also have deprioritized relevant studies, and it cannot be ruled out that some were missed. Nevertheless, subsequent snowball searches of the reference lists of included papers did not yield many eligible studies, suggesting that the screening process likely captured the most relevant evidence. Third, the reporting of intervention components in the included studies, particularly PSD features, was often unclear, making it difficult to extract and code these elements. Inadequate reporting is a well-known issue in digital health research, limiting comparison of interventions, identification of success factors, and adaptation across populations. Although reporting guidelines such as CONSORT-EHEALTH (Consolidated Standards of Reporting Trials of Electronic and Mobile Health Applications and Online Telehealth) [101] improve transparency, they do not explicitly cover feature-level or design-specific elements such as persuasive features. Consistent reporting of these aspects remains essential to ensure that knowledge about digital health interventions is generalizable and replicable. Despite these challenges, applying the PSD framework here provided a structured lens for analyzing intervention design and helped provide a more systematic understanding of how persuasive features are used in child health interventions. Fourth, although a clear-cut criterion was necessary to categorize interventions consistently and reproducibly into general population and low SEP groups, this approach also has limitations. For example, classifying interventions as targeting low SEP only when this was explicitly stated by study authors may have underrepresented or misclassified interventions delivered in implicitly disadvantaged contexts.

### Conclusion

In conclusion, this review contributes to the evidence base by summarizing digital and blended lifestyle interventions for preschool-aged children across multiple behavioral lifestyle domains, addressing the limited attention given to early childhood in digital health research. To our knowledge, it is also among the few reviews to compare interventions developed for the general population with those targeting families with low SEP, offering a descriptive perspective on similarities and differences across populations. Although only limited descriptive patterns emerged across intervention characteristics and effectiveness categories, the review nevertheless provides an overview of existing approaches and documents heterogeneity in characteristics and reporting. Moreover, it highlights areas where evidence remains sparse or unevenly distributed. These descriptive findings, combined with known barriers experienced by families with fewer resources, invite further reflection on how feasibility, acceptability, and contextual relevance may influence whether and how families are able to engage with interventions. Taken together, these insights point toward several directions for future work. low SEP families remain underrepresented in intervention development and evaluation, and greater attention is needed to understand feasibility, contextual fit, and acceptability from their perspectives. Approaches that consider multistakeholder and multilevel influences may better support families’ opportunities to adopt healthy lifestyle behaviors. Additionally, children themselves may need to be more directly involved in future intervention development. Sustainable and equitable progress may require complementing individual-focused strategies with broader structural and environmental supports. Future research could therefore examine how individual-level interventions can be embedded within wider community, service, and policy systems. Ultimately, advancing this field may depend on designing interventions that are contextually grounded, system-aware, and responsive to the diverse realities of families’ everyday lives.

## Supplementary material

10.2196/86596 Multimedia Appendix 1 Full electronic search strategies for all databases used in the scoping review.

10.2196/86596 Multimedia Appendix 2 List of papers used as prior knowledge to train ASReview (Utrecht University).

10.2196/86596 Multimedia Appendix 3 Overview of persuasive systems design features.

10.2196/86596 Multimedia Appendix 4 Overview of the characteristics of included studies, including authors, publication year, study design and aim, intervention name, and quality assessment.

10.2196/86596 Multimedia Appendix 5 Overview of the characteristics of included interventions, including population; intervention name and goal; target behaviors; target group; theories, frameworks, and evidence-based guidelines; strategy and channel; delivery environment; persuasive features; and effectiveness.

10.2196/86596 Multimedia Appendix 6 Screenshots of included interventions.

10.2196/86596 Multimedia Appendix 7 Overview of the critical appraisal of included randomized controlled trials.

10.2196/86596 Multimedia Appendix 8 Critical appraisal table for quasi-experimental studies.

10.2196/86596 Checklist 1 PRISMA-ScR checklist.

10.2196/86596 Checklist 2 PRISMA-S checklist.

## Abbreviations

AI: artificial intelligence
BCT: behavior change technique
CONSORT-EHEALTH: Consolidated Standards of Reporting Trials of Electronic and Mobile Health Applications and Online Telehealth
JBI: Joanna Briggs Institute
PRISMA-S: Preferred Reporting Items for Systematic Reviews and Meta-Analyses literature search extension
PRISMA-ScR: Preferred Reporting Items for Systematic Reviews and Meta-Analyses extension for Scoping Reviews
PSD: persuasive systems design
RCT: randomized controlled trial
SEP: socioeconomic position
WHO: World Health Organization
