# Supplementary material for: Digital and Blended Lifestyle Interventions for Preschool-Aged Children and Families With a Low Socioeconomic Position and the General Population: Scoping Review
Source: J Med Internet Res. 2026 Jun 5;28:e86596. doi: 10.2196/86596 (PMC13240985; doi:10.2196/86596)
Supplement: Multimedia Appendix 1 [file jmir-v28-e86596-s001.docx]

| Database | Field | Search String |
| --- | --- | --- |
| Scopus | Title, abstract, keywords | TITLE-ABS-KEY ("electronic health" OR "digital health" OR ehealth OR "e-health" OR mhealth OR "health information technolog*" OR "mobile health" OR "health technolog*" OR "social media" OR whatsapp OR facebook OR instagram OR tiktok OR blended) **AND** TITLE-ABS-KEY ("lifestyle intervention*" OR "health* lifestyle" OR "weight management" OR "weight loss" OR nutr* OR diet* OR obesity OR "physical* exercis*" OR "sedentary behaviour" OR "oral hygien*" OR "dental care" OR caries OR "oral health*" OR sleep OR breastfe* OR lactat* OR life-style) **AND** TITLE-ABS-KEY (child* OR infant* OR postpartum OR "pre-school" OR parent* OR famil* OR teacher* OR "school-based" OR caretaker* OR father OR mother OR "care professional*" OR "pregnant wom*" OR pregnan* OR maternal OR paternal OR toddler OR baby OR babies OR newborn OR new-born) |
| Web of Science | Topic | TS=("electronic health" OR "digital health" OR ehealth OR "e-health" OR mhealth OR "health information technolog*" OR "mobile health" OR "health technolog*" OR "social media" OR whatsapp OR facebook OR instagram OR tiktok OR blended) **AND** TS=("lifestyle intervention*" OR "health* lifestyle" OR "weight management" OR "weight loss" OR nutr* OR diet* OR obesity OR "physical* exercis*" OR "sedentary behaviour" OR "oral hygien*" OR "dental care" OR caries OR "oral health*" OR sleep OR breastfe* OR lactat* OR life-style) **AND** TS=(child* OR infant* OR postpartum OR "pre-school" OR parent* OR famil* OR teacher* OR "school-based" OR caretaker* OR father OR mother OR "care professional*" OR "pregnant wom*" OR pregnan* OR maternal OR paternal OR toddler OR baby OR babies OR newborn OR new-born) |
| ERIC | TX All Text | ("electronic health" OR "digital health" OR ehealth OR "e-health" OR mhealth OR "health information technolog*" OR "mobile health" OR "health technolog*" OR "social media" OR whatsapp OR facebook OR instagram OR tiktok OR blended) **AND** ("lifestyle intervention*" OR "health* lifestyle" OR "weight management" OR "weight loss" OR nutr* OR diet* OR obesity OR "physical* exercis*" OR "sedentary behaviour" OR "oral hygien*" OR "dental care" OR caries OR "oral health*" OR sleep OR breastfe* OR lactat* OR life-style) **AND** (child* OR infant* OR postpartum OR "pre-school" OR parent* OR famil* OR teacher* OR "school-based" OR caretaker* OR father OR mother OR "care professional*" OR "pregnant wom*" OR pregnan* OR maternal OR paternal OR toddler OR baby OR babies OR newborn OR new-born) |
| ACM Digital Library | Abstract | *[[Abstract: "electronic health"] OR [Abstract: "digital health"] OR [Abstract: ehealth] OR [Abstract: "e-health"] OR [Abstract: mhealth] OR [Abstract: "health information technolog*"] OR [Abstract: "mobile health"] OR [Abstract: "health technolog*"] OR [Abstract: "social media"] OR [Abstract: whatsapp] OR [Abstract: facebook] OR [Abstract: instagram] OR [Abstract: tiktok] OR [Abstract: blended]] AND [[Abstract: "lifestyle intervention*"] OR [Abstract: "health* lifestyle"] OR [Abstract: "weight management"] OR [Abstract: "weight loss"] OR [Abstract: nutr*] OR [Abstract: diet*] OR [Abstract: obesity] OR [Abstract: "physical* exercis*"] OR [Abstract: "sedentary behaviour"] OR [Abstract: "oral hygien*"] OR [Abstract: "dental care"] OR [Abstract: caries] OR [Abstract: "oral health*"] OR [Abstract: sleep] OR [Abstract: breastfe*] OR [Abstract: lactat*] OR [Abstract: life-style]] AND [[Abstract: child*] OR [Abstract: infant*] OR [Abstract: postpartum] OR [Abstract: "pre-school"] OR [Abstract: parent*] OR [Abstract: famil*] OR [Abstract: teacher*] OR [Abstract: "school-based"] OR [Abstract: caretaker*] OR [Abstract: father] OR [Abstract: mother] OR [Abstract: "care professional*"] OR [Abstract: "pregnant wom*"] OR [Abstract: pregnan*] OR [Abstract: maternal] OR [Abstract: paternal] OR [Abstract: toddler] OR [Abstract: baby] OR [Abstract: babies] OR [Abstract: newborn] OR [Abstract: new-born]]* |
| Cochrane library | Title, abstract, keywords | "electronic health" OR "digital health" OR ehealth OR "e-health" OR mhealth OR (health information NEXT technolog*) OR "mobile health" OR (health NEXT technolog*) OR "social media" OR whatsapp OR facebook OR instagram OR tiktok OR blended in Title Abstract Keyword **AND** (lifestyle NEXT intervention*) OR (health* NEXT lifestyle) OR "weight management" OR "weight loss" OR nutr* OR diet* OR obesity OR (physical* NEXT exercis*) OR "sedentary behaviour" OR (oral NEXT hygien*) OR "dental care" OR caries OR (oral NEXT health*) OR sleep OR breastfe* OR lactat* OR life-style in Title Abstract Keyword **AND** child* OR infant* OR postpartum OR "pre-school" OR parent* OR famil* OR teacher* OR "school-based" OR caretaker* OR father OR mother OR (care NEXT professional*) OR (pregnant NEXT wom*) OR pregnan* OR maternal OR paternal OR toddler OR baby OR babies OR newborn OR “new-born” in Title Abstract Keyword |
