# Supplementary material for: Digital and Blended Lifestyle Interventions for Preschool-Aged Children and Families With a Low Socioeconomic Position and the General Population: Scoping Review
Source: J Med Internet Res. 2026 Jun 5;28:e86596. doi: 10.2196/86596 (PMC13240985; doi:10.2196/86596)
Supplement: Multimedia Appendix 2 [file jmir-v28-e86596-s002.docx]

**Papers given to ASReview as relevant:**

1. Ihab M, Abdelaziz WEE-D, Hassan W, El Tantawi M. Development and acceptability of behavioral interventions promoting mothers’ brushing of pre-school children’s teeth: The preparation phase of the multi-phase optimization strategy framework. BMC Oral Health 2023 Aug 31;23(1):616. doi: [10.1186/s12903-023-03351-x](https://doi.org/10.1186/s12903-023-03351-x)

2. Markides BR, Hesketh KD, Maddison R, Laws R, Denney-Wilson E, Campbell KJ. Fussy Eating Rescue, a mobile-web app for responsive feeding practises among parents of toddlers: protocol for a pilot randomised controlled feasibility trial. Pilot Feasibility Stud 2023 July 22;9(1):128. doi: [10.1186/s40814-023-01278-2](https://doi.org/10.1186/s40814-023-01278-2)

3. Nyström CD, Sandin S, Henriksson P, Henriksson H, Trolle-Lagerros Y, Larsson C, Maddison R, Ortega FB, Pomeroy J, Ruiz JR, Silfvernagel K, Timpka T, Löf M. Mobile-based intervention intended to stop obesity in preschool-aged children: the MINISTOP randomized controlled trial ,. The American Journal of Clinical Nutrition 2017 June;105(6):1327–1335. doi: [10.3945/ajcn.116.150995](https://doi.org/10.3945/ajcn.116.150995)

4. Hojati A, Alesaeidi S, Izadi S, Nikniaz A, Farhangi MA. MyKid’sNutrition mobile application trial: a randomized controlled trial to promote mothers’ nutritional knowledge and nutritional status of preschool children with undernutrition—a study protocol. Trials 2023 Aug 19;24(1):544. doi: [10.1186/s13063-023-07503-w](https://doi.org/10.1186/s13063-023-07503-w)

5. Zhang Z, Li L, Li X, Okely A. Promoting healthy sleep in Chinese kindergarteners through a family-based intervention: protocol of the “Healthy Sleep” randomised controlled trial. BMC Public Health 2023 Sept 26;23(1):1865. doi: [10.1186/s12889-023-16806-1](https://doi.org/10.1186/s12889-023-16806-1)

6. Perdew M, Liu S, Rhodes R, Ball GDC, Mâsse LC, Hartrick T, Strange K, Naylor P-J. The Effectiveness of a Blended In-Person and Online Family-Based Childhood Obesity Management Program. Childhood Obesity 2021 Jan 1;17(1):58–67. doi: [10.1089/chi.2020.0236](https://doi.org/10.1089/chi.2020.0236)

**Papers given to ASReview as irrelevant:**

1. Al-Mardini M, Aloul F, Sagahyroon A, Al-Husseini L. Classifying obstructive sleep apnea using smartphones. Journal of Biomedical Informatics 2014 Dec;52:251–259. doi: [10.1016/j.jbi.2014.07.004](https://doi.org/10.1016/j.jbi.2014.07.004)

2. Bhattarai S, Yadav SK, Thapaliya B, Giri S, Bhattarai B, Sapkota S, Manandhar S, Arjyal A, Saville N, Harris-Fry H, Haghparast-Bidgoli H, Copas A, Hillman S, Baral SC, Morrison J. Contextual factors affecting the implementation of an anemia focused virtual counseling intervention for pregnant women in plains Nepal: a mixed methods process evaluation. BMC Public Health 2023 July 6;23(1):1301. doi: [10.1186/s12889-023-16195-5](https://doi.org/10.1186/s12889-023-16195-5)

3. Tjeertes J, Bacino CA, Bichell TJ, Bird LM, Bustamante M, Crean R, Jeste S, Komorowski RW, Krishnan ML, Miller MT, Nobbs D, Ochoa-Lubinoff C, Parkerson KA, Rotenberg A, Sadhwani A, Shen MD, Squassante L, Tan W-H, Vincenzi B, Wheeler AC, Hipp JF, Berry-Kravis E. Enabling endpoint development for interventional clinical trials in individuals with Angelman syndrome: a prospective, longitudinal, observational clinical study (FREESIAS). J Neurodevelop Disord 2023 July 26;15(1):22. doi: [10.1186/s11689-023-09494-w](https://doi.org/10.1186/s11689-023-09494-w)

4. Pritchard E, Van Vreden C, Xia T, Newnam S, Collie A, Lubman DI, De Almeida Neto A, Iles R. Impact of work and coping factors on mental health: Australian truck drivers’ perspective. BMC Public Health 2023 June 6;23(1):1090. doi: [10.1186/s12889-023-15877-4](https://doi.org/10.1186/s12889-023-15877-4)

5. Mzembe T, Chikwapulo V, Kamninga TM, Vellemu R, Mohamed S, Nthakomwa L, Chifungo C, Wazny K, Musau K, Abdullahi L, Peterson M, Madise N, Chipeta MG. Interventions to enhance healthcare utilisation among pregnant women to reduce maternal mortality in low- and middle-income countries: a review of systematic reviews. BMC Public Health 2023 Sept 6;23(1):1734. doi: [10.1186/s12889-023-16558-y](https://doi.org/10.1186/s12889-023-16558-y)

6. Safavi A, Lin Y, Skarsgard ED. Perinatal management of congenital diaphragmatic hernia: when and how should babies be delivered? Results from the Canadian Pediatric Surgery Network. Journal of Pediatric Surgery 2010 Dec;45(12):2334–2339. doi: [10.1016/j.jpedsurg.2010.08.026](https://doi.org/10.1016/j.jpedsurg.2010.08.026)

7. Chambers Iv E, Maughan C, Padmanabhan N, Alavi S, Adedji A. Sensory analysis of 20% solids fortified blended porridge. BFJ 2019 Feb 4;121(2):633–641. doi: [10.1108/BFJ-05-2018-0280](https://doi.org/10.1108/BFJ-05-2018-0280)

8. Chalermchutidej W, Manaboriboon B, Sanpawitayakul G, Theppiban S, In-iw S. Sleep, social media use and mental health in female adolescents aged 12 to 18 years old during the COVID-19 pandemic. BMC Pediatr 2023 Aug 14;23(1):398. doi: [10.1186/s12887-023-04218-4](https://doi.org/10.1186/s12887-023-04218-4)
