# Supplementary material for: Digital and Blended Lifestyle Interventions for Preschool-Aged Children and Families With a Low Socioeconomic Position and the General Population: Scoping Review
Source: J Med Internet Res. 2026 Jun 5;28:e86596. doi: 10.2196/86596 (PMC13240985; doi:10.2196/86596)
Supplement: Multimedia Appendix 3 [file jmir-v28-e86596-s003.docx]

| Primary Task Support | Dialogue Support | System Credibility Support | Social Support |
| --- | --- | --- | --- |
| Reduction  *Smoking cessation Web site provides an interactive test that measures how much money a user will save with quitting.* | Praise  *Mobile application that aims at motivating teenagers to exercise praises user by sending automated text-messages for reaching individual goals.* | Trustworthiness  *Company Web site provides information related to its products rather than simply providing biased advertising or marketing information.* | Social learning  *A shared fitness journal in a mobile application for encouraging physical activity.* |
| Tunneling  *Smoking cessation Web site offers information about treatment opportunities after a user has taken an interactive test about how addicted (s)he is on tobacco.* | Rewards  *Heart rate monitor gives users a virtual trophy if they follow their fitness program.* | Expertise  *Mobile application is updated regularly and there are no dangling links or out-of-date information.* | Social comparison  *Users can share and compare information related to their physical health and smoking behavior via instant messaging application.* |
| Tailoring  *Personal trainer Web site provides different information content for different user groups, e.g. beginners and professionals.* | Reminders  *Caloric balance monitoring application sends text-messages to its users as daily reminders.* | Surface credibility  *There are only a limited number of, and a logical reason for, ads on a Web site or mobile application.* | Normative influence  *A smoking cessation application shows pictures of newborn babies with serious health problems due to the mother’s smoking habit.* |
| Personalization  *Arguments most likely to be relevant for the user presented first on a professional Web site rather than in random order.* | Suggestion  *Application for healthier eating habits suggests that children eat fruits instead of candy at snack time.* | Real-world feel  *Company Web site provides possibilities to contact specific people through sending feedback or asking questions.* | Social facilitation  *Users of a computer-based learning environment can recognize how many co-students are doing their assigned homework at the same time as them.* |
| Self-monitoring  *Heart rate monitor presents a user’s heart rate and the duration of the exercise.* | Similarity  *Slang names are used in an application which aims at motivating teenagers to exercise.* | Authority  *Web site quotes an authority, such as a statement by government health office.* | Cooperation  *The behavioral patterns of overweight patients are studied through a mobile application, which collects data and sends it to a central server where it can be analyzed at the group level in more detail.* |
| Simulation  *Before-and-after pictures of people who have lost weight are presented on a Web site.* | Liking  *Web site that aims at encouraging children to take care of their pets properly has pictures of cute animals.* | Third-party endorsements  *E-shop shows a logo of a certificate that assures that they use secure connections.* | Competition  *Online competition, such as Quit and Win (stop smoking for a month and win a prize).* |
| Rehearsal  *A flying simulator to help flight pilots practice for severe weather conditions.* | Social role  *E-health application has a virtual specialist to support communication between users and health specialists.* | Verifiability  *Claims on a Web site are supported by offering links to other web sites.* | Recognition  *Names of awarded people, such as “stopper of the month,” are published on a Web site.* |

**Note.** Examples reproduced from Oinas-Kukkonen and Harjumaa [1] under the Creative Commons Attribution License (CC BY 4.0).

**References (for Multimedia Appendix 3)**

1. Oinas-Kukkonen H, Harjumaa M. Persuasive Systems Design: Key Issues, Process Model, and System Features. Communications of the Association for Information Systems. 2009;24. doi: <https://doi.org/10.17705/1CAIS.02428>.
