# Supplementary material for: Digital and Blended Lifestyle Interventions for Preschool-Aged Children and Families With a Low Socioeconomic Position and the General Population: Scoping Review
Source: J Med Internet Res. 2026 Jun 5;28:e86596. doi: 10.2196/86596 (PMC13240985; doi:10.2196/86596)
Supplement: Multimedia Appendix 4 [file jmir-v28-e86596-s004.docx]

| **Authors, date of publication** | **Study design and goal** | **Intervention name** | **Quality assessment** |
| --- | --- | --- | --- |
| Aguirre et al.  2019 [1] | Protocol  To describe a single-blinded and parallel RCT to analyze the effectiveness of educational mobile messages as an adjuvant strategy for the prevention of ECC. | Not named | n. a. |
| Alexandrou et al.  2023a [2] | Hybrid type 1 effectiveness implementation trial  To evaluate the real-world effectiveness of a 6-month mHealth intervention on children’s intake of fruits, vegetables, sweet and savory treats, sweet drinks, moderate-to-vigorous physical activity, and screen time, and on parental self-efficacy (PSE) for promoting healthy lifestyle behaviors, and children’s body mass index (BMI). | MINISTOP 2.0 | Medium risk of bias |
| Alexandrou et al.  2023b [3] | Acceptability & Feasibility Interview Study  To explore and describe user experiences, acceptability and feasibility of the intervention in parents and Swedish child healthcare nurses. | MINISTOP 2.0 | n. a. |
| Ball et al.  2020 [4] | Protocol  To describe the methods for implementing and assessing the effectiveness and end user acceptability of the intervention. | PICNIC | n. a. |
| Beck et al.  2023 [5] | RCT  To evaluate the impact of the intervention on infant feeding, screen time, and sleep practices and to use qualitative methods to explore mechanisms of action. | Futuros Fuertes | Medium risk of bias |
| Beckerman et al.  2019 [6] | Protocol  To describe how theory, implementation science principles, and CBPR methods informed CHL content and implementation strategies, and outline the evaluation design, measures, and analytic plan for the trial. | Communities for Healthy Living (CHL) | n. a. |
| Bellows et al.  2025 [7] | Protocol  To outlines the 3 phases of design, development, and testing of the eHEROs mobile app: (1) formative research, intervention mapping, app development and testing, and pilot testing | eHEROs | n. a. |
| Billah et al.  2021 [8] | Parallel five-arm cRCT  To assess the impact of nutrition counselling delivered via a digital job aid on dietary diversity of children aged 6–23 months using data from a cRCT in Bangladesh. | Not named | Low risk of bias |
| Blomkvist et al.  2018 [9] | Protocol  To describe the design and methodology of the cRCT aimed to develop, measure and compare the effect of two different interventions among one-year-old children in kindergartens to reduce food neophobia and promote healthy diets. | Barns matmot 2.0 | n. a. |
| Blomkvist et al.  2021 [10] | cRCT  To assess the effect of a cRCT amongst 1-year-old children in kindergarten to reduce food neophobia and promote healthy eating . | Barns matmot 2.0 | Medium risk of bias |
| Branco dos Santos Lima & Pinheiro Barbosa  2025[11] | Parallel RCT  To evaluate the effectiveness of a web-based educational intervention, grounded in self-efficacy theory, on the sleep quality of infants aged 10-24 months by reducing nighttime awakenings and increasing total nighttime sleep duration. | Not named | Medium risk of bias |
| Campbell et al.  2016 [12] | Protocol  To describe the design and methodology of the cRCT to test the efficacy of an extended (33 versus 15 month) and enhanced (use of web-based materials, and Facebook® engagement), version of the original Melbourne InFANT Program intervention in a new cohort. | InFANT Extend Program | n. a. |
| Chen et al.  2023 [13] | Protocol  To describe the design and evaluation plan for a cRCT to assess the impact of the Healthy Future programme on maternal and child health in rural China through a cluster randomised controlled trial. | Healthy Future Programme | n. a. |
| Chen et al.  2025 [14] | Acceptability Study  To examine the acceptance and satisfaction of the intervention among participating parents and daycare teachers. | Happy Family, Healthy Kids | n. a. |
| Choonhawarakorn et al.  2024 [15] | RCT  To evaluate the effects of teledentistry in the form of a message service (MS) via the social media LINE on the promotion of healthy oral health behaviours that contribute to ECC prevention in infants and toddlers among a group of Thai caregivers. | Not named | Medium Risk of Bias |
| Delisle et al.  2015 [16] | Protocol  To report the study design and methodology of the trial to determine the effectiveness of a 6-month mHealth parental intervention on body fatness, dietary habits, physical fitness, physical activity and sedentary behavior in 4-year-olds. | MINISTOP (Mobile-Based Intervention Intended to Stop Obesity in Preschoolers) | n. a. |
| Dennis et al.  2021 [17] | Protocol  To describe the design and methodology of a randomised controlled multicentre trial to determine whether a 4-phase ‘preconception to early childhood’ lifecourse intervention can reduce the rate of child overweight and obesity. | Healthy Life Trajectory Initiative (HeLTI) Canada | n. a. |
| Downing et al.  2017 [18] | Protocol  To describe a two-armed, pilot RCT to evaluate the feasibility and efficacy of a parent-focused, predominantly mobile phone-delivered intervention to reduce sedentary behaviour in 2- to 4-year-old children. | Mini Movers | n. a. |
| Downing et al.  2018 [19] | Two-arm pilot randomized controlled trial  To evaluate a parent-focused, predominantly text message–delivered intervention to reduce sedentary behavior in 2- to 4-year-old children. | Mini Movers | Low risk of bias |
| Downs et al.  2019 [20] | Pilot study  To design an mHealth voice messaging intervention aimed at improving IYCF practices and examine its implementation and impact in households with children 6-23 months in three rural villages in Senegal. | Not named | Low risk of bias |
| Downs et al.  2023 [21] | Protocol  To describe the design and methodology of a cRCT to determine the impact of an mHealth IYCF intervention on IYCF practices and nutrition outcomes; and to examine the implementation, costs, and opportunities for scaling up the mHealth messaging intervention. | IIMAANJE | n. a. |
| Ferdous et al.  2024 [22] | Feasibility Study  To describe the design and development of the intervention delivery system; to present the adequacy of intervention visit coverage from real-time data; and to explore the facilitating and challenging issues of this system described by users (CHWs and their supervisors). | Not named | n. a. |
| Garcia et al.  2024 [23] | Protocol  To describe the design and methodology of the cRCT to experimentally test the relative effectiveness and costs of a traditional in-person delivery model against a hybrid model that combines in-person meetings with remote mHealth delivery. | Msingi Bora | n. a. |
| Gago et al.  2023 [24] | Cluster randomized family-centered obesity prevention trial  To evaluate whether children in the intervention vs. the control experienced greater improvements in Body Mass Index z-score (BMIz) and weight-related behaviors. | Communities for Healthy Living (CHL) | Low risk of bias |
| Ghofranipour et al.  2022 [25] | Pre and post-pilot study  To design and evaluate a behaviour change intervention programme to promote weight management in preschool children of Tehran with any weight status. | Preschooler's weight management promotion, (PWMP) | Medium risk of bias |
| Hammersley et al.  2017 [26] | Protocol  To describe the design and methodology of a RCT to determine the efficacy of *Time2bHealthy* - an online healthy lifestyle program for parents of preschool-aged children. | Time2bHealthy | n.a. |
| Hammersley et al.  2019 [27] | RCT  To assess the efficacy of a parent-focused, internet-based healthy lifestyle program for preschool-aged children, who are overweight or at or above the fiftieth percentile for body mass index (BMI) for their age and sex, on child BMI, obesity-related behaviors, parent modeling, and parent self-efficacy. | Time2bHealthy | Low risk of bias |
| He et al.  2024 [28] | cRCT  To evaluate the feasibility and potential efficacy of a family-based PA intervention on objectively measured PA, fundamental movement skills, parental efficacy, support, goal setting and parent–child co-activity. | Not named | Medium Risk of Bias |
| Heerman et al.  2022 [29] | Protocol  To detail the methods for a multi-site, comparative effectiveness trial to compare the effectiveness of two approaches to childhood obesity prevention in the first two years of life. | Greenlight Plus | n. a. |
| Heerman et al.  2024 [30] | Multicenter, individually randomized, parallel-group trial  To test the effect of adding a digital obesity prevention intervention to health behavior counseling delivered by pediatric primary care clinicians. | Greenlight Plus | Medium Risk of Bias |
| Helle et al.  2017 [31] | Protocol  To describe the design and methodology of a RCT to evaluate an Internet-based tool for parents of children between 6 and 12 months. | barnE-mat (in Early Food for Future Health) | n. a. |
| Helle et al.  2019a [32] | RCT  To evaluate the effects of an eHealth intervention on parental feeding practices and infant eating at child age 12 months. | barnE-mat | Medium risk of bias |
| Helle et al.  2019b [33] | RCT  To evaluate the effects of the intervention at child age 24 months, one year after cessation. | barnE-mat | Medium risk of bias |
| Henriksson et al.  2020 [34] | Protocol  To describe the study design and methodology of a trial to evaluate whether a parent-oriented mobile health app integrated into primary child health care can improve diet and physical activity behaviours and reduce the prevalence of overweight and obesity in preschool-aged children as well as to evaluate the implementation among child health care nurses and parents. | MINISTOP 2.0 | n. a. |
| Hesketh et al.  2022 [35] | Protocol  To describe the design and methodology of a RCT to assess the efficacy of the mHealth intervention in 2-year-old children at conclusion of the 12-month intervention. | Let's Grow | n. a. |
| Ihab et al.  2022 [36] | Protocol  To describe the design and methodology of a randomized factorial trial to develop an optimized behavior modification intervention to increase parents’ brushing of their pre-school children’s teeth using the multi-phase optimization strategy (MOST). | Not named | n. a. |
| Ihab et al.  2023 [37] | Feasibility pilot study  To describe the development and assesses the acceptability of 3 intervention components using MOST to promote mothers’ brushing of their preschool children’s teeth. | Not named | n. a. |
| Ihab et al.  2024 [38] | Factorial optimization RCT  To test the effects of 3 components using a full factorial optimization RCT, evaluating the main effects and 2- and 3-way interactions on reduced plaque and brushing of children’s teeth after 3 months, and to determine the optimal combination of components. | Not named | Medium Risk of Bias |
| Jiying et al.  2024 [39] | One-group, quasi-experimental pilot study  To evaluate the preliminary effects of a healthy eating and stress management program targeting multiple theoretical variables on improving eating behavior, food insecurity, anthropometric characteristics, cardiovascular health, and mental well-being. | Happy Family, Healthy Kids | Medium Risk of Bias |
| Karssen et al.  2021 [40] | Protocol  To describe the application of the Intervention Mapping Protocol (IMP) for the development of an app-based preventive intervention program to promote healthy energy balance–related parenting practices among parents of children with a lower SEP. | Samen Happie! | n. a. |
| Karrsen et al.  2022 [41] | Randomized controlled intervention study  To examine the effectiveness of the app-based program in terms of reach, use, acceptability, and child zBMI among Dutch parents and infants. | Samen Happie! | Low risk of bias |
| Kitsaras et al.  2020 [42] | Protocol  Describes the development and methodology of an early phase study | BRIC | n. a. |
| Lafave et al.  2024 [43] | Protocol  To present the design of the CHEERS eHealth program to improve nutrition and physical activity practices within Early Childhood Education and Care (ECEC) centers. | CHEERS | n. a. |
| Lee et al.  2023 [44] | Pilot randomized control and intervention study  To evaluate a theory-based, multicomponent eHealth intervention aimed at improving child health behaviors and parental psychosocial attributes and feeding practices. | Not named | Medium risk of bias |
| Lewis et al.  2023 [45] | Pilot parallel group RCT  To assess the potential feasibility and efficacy of the newly developed intervention. | Ready, Set, Gulp! | Low risk of bias |
| Ling et al.  2024 [46] | cRCT  To evaluate the preliminary efficacy of the intervention vs. control on preschoolers' proximal behavioural changes of moderate-to-vigorous PA, light PA, diet quality, and screen time; and distal anthropometric outcomes. | FirstStep2Health | Medium Risk of Bias |
| Ling et al.  2025 [47] | One-group, pre-post feasibility study  To examine feasibility, acceptability, and satisfaction of a 5-week mindfulness-based lifestyle intervention among preschoolers, caregivers, and childcare teachers. | Not named | n.a. |
| Lotto et al.  2020 [48] | Single-blind, 2-parallel arm RCT  To evaluate the effectiveness of oral health educational text messages to aid in the control of early childhood caries in low socioeconomic children, considering caries experience, dental biofilm, and dietary habits. | Not named | Low risk of bias |
| Lozada-Tequeanes et al.  2024 [49] | Effectiveness-implementation hybrid trial  To assess the effectiveness and implementation of an mHealth intervention in promoting physical activity and healthy feeding among Mexican PCs of children under 5 years. | NUTRES | Medium Risk of Bias |
| Markides et al.  2023 [50] | Protocol  To describe a randomised controlled feasibility pilot aiming to evaluate Fussy Eating Rescue, to investigate feasibility and acceptability, and to explore indications of intervention effect on parents’ feeding practises or children’s eating behaviours. | Fussy Eating Rescue | n. a. |
| Militello et al.  2016 [51] | One-group pre and posttest preexperimental design  To establish the feasibility, acceptability, and preliminary effects of a 7-session cognitive behavioral intervention combined with tailored and adaptive SMS regarding healthy lifestyle beliefs, perceived difficulty, and behaviors of parents of overweight and obese (OW/OB) preschoolers (aged 3-5 years) delivered in a primary care setting. | TEXT2COPE | Low risk of bias |
| Nezami et al.  2016 [52] | Protocol  To describe the study design and intervention seeking to test the efficacy of a 6-month primarily smartphone-delivered program to reduce sugar-sweetened beverage and juice consumption among children ages 3–5 whose mothers are overweight or obese. | Smart Moms | n. a. |
| Nezami et al.  2017 [53] | RCT  To test the efficacy of a smartphone-delivered intervention to reduce parent-provided sugar-sweetened beverage and juice (SSB/juice) consumption among children ages 3–5 and maternal weight. | Smart Moms | Low risk of bias |
| Nyström et al.  2017 [54] | RCT  To assess the effectiveness of a mobile health (mHealth) obesity prevention program on body fat, dietary habits, and physical activity in healthy Swedish children aged 4.5 y. | MINISTOP (Mobile-Based Intervention Intended to Stop Obesity in Preschoolers) | Low risk of bias |
| Nyström et al. (2)  2018 [55] | RCT  To investigate if the intervention 12-months after baseline measurements: (i) improved FMI and (ii) had a maintained effect on a composite score comprised of FMI and dietary and physical activity variables. | MINISTOP (Mobile-Based Intervention Intended to Stop Obesity in Preschoolers) | Low risk of bias |
| Øverby et al.  2023 [56] | Protocol  To describe the design and methodology of the hybrid type 1 mixed methods implementation study seeking to test the efficacy of a 6-month primarily smartphone-delivered program to reduce sugar-sweetened beverage and juice consumption among children ages 3–5 whose mothers are overweight or obese. | Nutrition now | n. a. |
| Pakarinen et al.  2018 [57] | Protocol  To describe the design and methodology of a RCT aiming to evaluate the effect of the digital WellWe intervention on parental self-efficacy for healthy behaviours, mindfulness in parenting and a family-centred approach in the extensive health examinations of 4-year-old children. | WellWe | n. a. |
| Peden et al.  2022 [58] | Stepped-wedge clustered randomized controlled trial  To evaluate the efficacy of a ‘blended’ professional learning program for early childhood educators on the physical activity and healthy eating environments and policies and on the physical activity levels of children in ECEC services. | HOPPEL (Healthy Online Professional Program for Early Learners) | Low risk of bias |
| Po'e et al.  2013 [59] | Protocol  To describe the design and methodology of the parallel-group, RCT designed to evaluate the efficacy of a family-centered, behavioral intervention to prevent pediatric obesity (i.e., BMI trajectory) among children ages 3–5. | The Growing Right Onto Wellness (GROW) | n. a. |
| Raat et al.  2013 [60] | Protocol  To describe the design of a cRCT aiming to assess the effects of the two overweight prevention interventions with regard to child health behaviors and child Body Mass Index. | E-health4Uth Healthy toddler | n. a. |
| Ramírez et al.  2025 [61] | Intervention design study  To describe the development of a theory-informed intervention and, during this phase, to evaluate the acceptability and usability among the intended audience. | FUNS (Familias Unidas, Niños Sano) | n. a. |
| Riera-Navarro et al.  2024 [62] | Protocol  To describe the design and methodology of the RCT aiming to assess the effects of the two overweight prevention interventions with regard to child health behaviors and child Body Mass Index. | feediNg gUidelines infanT RandomIsEd coNtrolled Trial (NutrienT) | n. a. |
| Røed et al.  2019 [63] | Protocol  To describe the rationale, development, and evaluation design of a RCT to develop and evaluate the effect of an eHealth intervention called Food4toddlers. | Food4toddlers | n. a. |
| Røed et al.  2021 [64] | RCT  To examine the effect of a parent-focused eHealth intervention on the child’s diet assessed at two time points postintervention. | Food4toddlers | Medium risk of bias |
| Sandborg et al.  2025 [65] | RCT  To investigate the effect of a digital intervention, intended to help parents promote healthy movement behaviours in toddlers on the intermediary outcomes of parenting practices and cognitions and child skill development. | Let’s Grow | High risk of bias |
| Seyyedi et al.  2020 [66] | RCT  To assess the effect of a smartphone-based maternal nutritional education program for the complementary feeding of undernourished children under 3 years of age in a food-secure middle-income community in Urmia, Iran. | Not named | Low risk of bias |
| Thomas et al.  2024 [67] | Protocol  To describe the methodology of the MINISTOP 3.0 trial seeking to: (i) compare two different implementation strategies for MINISTOP 3.0 (Basic vs. Enhanced) on: acceptability, appropriateness, feasibility, organizational readiness to implement MINISTOP 3.0 within Swedish child healthcare (primary outcomes) as well as reach, costs, and adoption of MINISTOP 3.0 (secondary outcomes); (ii) evaluate cost-effectiveness of MINISTOP 3.0; (iii) explore the sustainability of MINISTOP 3.0; (iv) evaluate the determinants of effectiveness of MINISTOP 3.0 on children’s key lifestyle behaviours; and (v) investigate the long-term effects of MINISTOP 3.0 on children’s body mass index. | MINISTOP 3.0 | n. a. |
| Tomayko et al.  2017 [68] | Protocol  To describe the collaborative development of a RCT aiming to assess the effect of the intervention on fruit and vegetable intake, sugar intake, physical activity, TV/screen time, and stress and sleep. | Healthy Children, Strong Families 2 | n. a. |
| Tomayko et al.  2019 [69] | RCT  To test the efficacy of the expanded intervention to mitigate obesity risk in both urban and rural American Indian families. | Healthy Children, Strong Families 2 | Low risk of bias |
| van Grieken et al.  2017 [70] | Cluster RCT  To compare the effects of applying the intervention versus usual care (control) by assessing the following primary outcomes: breakfast daily, activity and outdoor play, sweetened beverages, screen time (ie, television (TV) watching and/or computer use), BMI, and the prevalence of overweight/obesity. | E-health4Uth Healthy toddler | Medium risk of bias |
| Wang et al.  2022 [71] | Protocol  To describe the design and methodology of a RCT to investigate the effectiveness of the HBM-based behavioral intervention using SMS to promote parental oral health care behaviors (toothbrushing and sugar intake control) and reduce ECC compared to conventional oral health education. | Not named | n. a. |
| Willis et al.  2023 [72] | Protocol  To present the background, rationale, and design to be used in the cRCT aiming to assess if a comprehensive early childhood obesity prevention initiative that also addresses the health of ECE workers elicits meaningful change in the ECE environment and health behaviors of children in their care. | Go NAPSACC | n. a. |
| Wu et al.  2023 [73] | Two-armed cluster randomized mutually controlled trial  To evaluate the effectiveness of a WeChat-based self-assessment with a tailored feedback report on improving complementary feeding and movement behaviour of children. | Not named | Medium risk of bias |
| Yoong et al.  2025 [74] | Protocol  To describe the design and methodology of a RCT to assess the efficacy of an 18-month digital health intervention (TinyBites) delivered to ECEC services and primary caregivers of children aged 4 to < 12 months on child age-adjusted and sex-adjusted body mass index-for-age z-score (zBMI) relative to usual care control in the Hunter New England (HNE) region of New South Wales, Australia. | TinyBites | n.a. |
| Yoshizaki et al.  2020 [75] | Single-arm pilot usability/feasibility study  To describe the app’s developmental design; check the system’s operation, acceptability, and usability; and determine the overall potential of the app to change infants’ sleep habits or parental cognition and behavior via user feedback in a small trial. | Nenne Navi | n. a. |
| Yoshizaki et al.  2023 [76] | RCT  To examine the app’s long-term continuity and effectiveness in improving children’s sleep habits and development and parental cognition and behavior. | Nenne Navi | High risk of bias |
| Zhang et al.  2021 [77] | Single group pre–post design  To evaluate the food-related behaviors and outcomes among new followers of the CM Facebook page who were low-income caregivers of children age 5 or younger. | Cooking Matters | Low risk of bias |

**References (for Multimedia Appendix 4)**

1. Aguirre PEA, Lotto M, Strieder AP, Cruvinel AFP, Cruvinel T. The Effectiveness of Educational Mobile Messages for Assisting in the Prevention of Early Childhood Caries: Protocol for a Randomized Controlled Trial. JMIR Res Protoc 2019 Sept 3;8(9):e13656. doi: 10.2196/13656

2. Alexandrou C, Henriksson H, Henström M, Henriksson P, Delisle Nyström C, Bendtsen M, Löf M. Effectiveness of a Smartphone App (MINISTOP 2.0) integrated in primary child health care to promote healthy diet and physical activity behaviors and prevent obesity in preschool-aged children: randomized controlled trial. Int J Behav Nutr Phys Act 2023 Feb 21;20(1):22. doi: 10.1186/s12966-023-01405-5

3. Alexandrou C, Rutberg S, Johansson L, Lindqvist A-K, Müssener U, Löf M. User experiences of an app-based mHealth intervention (MINISTOP 2.0) integrated in Swedish primary child healthcare among Swedish-, Somali- and Arabic-speaking parents and child healthcare nurses: A qualitative study. DIGITAL HEALTH 2023 Jan;9:20552076231203630. doi: 10.1177/20552076231203630

4. Ball R, Vaschak R, Bailey A, Whiteford G, Burrows T, Duncanson K, Collins C. Study Protocol of the Parents in Child Nutrition Informing Community (PICNIC) Peer Education Cohort Study to Improve Child Feeding and Dietary Intake of Children Aged Six Months to Three Years Old. Children 2019 Dec 27;7(1):3. doi: 10.3390/children7010003

5. Beck AL, Mora R, Joseph G, Perrin E, Cabana M, Schickedanz A, Fernandez A. A Multimethod Evaluation of the Futuros Fuertes Intervention to Promote Healthy Feeding, Screen Time, and Sleep Practices. Academic Pediatrics 2023 Sept;23(7):1351–1360. doi: 10.1016/j.acap.2023.05.006

6. Beckerman JP, Aftosmes-Tobio A, Kitos N, Jurkowski JM, Lansburg K, Kazik C, Gavarkovs A, Vigilante A, Kalyoncu B, Figueroa R, Klabunde R, Barouch R, Haneuse S, Taveras E, Davison KK. Communities for healthy living (CHL) – A family-centered childhood obesity prevention program integrated into Head Start services: Study protocol for a pragmatic cluster randomized trial. Contemporary Clinical Trials 2019 Mar;78:34–45. doi: 10.1016/j.cct.2019.01.002

7. Bellows LL, Oke S, Reyes LI, Carmona BA, Johnson SL. Development of a Digital Parent Intervention to Promote Healthy Eating and Activity in Preschoolers: The eHEROs Study. Journal of Nutrition Education and Behavior 2025 May;57(5):450–459. doi: 10.1016/j.jneb.2025.01.008

8. Billah SM, Ferdous TE, Kelly P, Raynes‐Greenow C, Siddique AB, Choudhury N, Ahmed T, Gillespie S, Hoddinott J, Menon P, Dibley MJ, Arifeen SE. Effect of nutrition counselling with a digital job aid on child dietary diversity: Analysis of secondary outcomes from a cluster randomised controlled trial in rural Bangladesh. Maternal & Child Nutrition 2022 Jan;18(1):e13267. doi: 10.1111/mcn.13267

9. Blomkvist EAM, Helland SH, Hillesund ER, Øverby NC. A cluster randomized web-based intervention trial to reduce food neophobia and promote healthy diets among one-year-old children in kindergarten: study protocol. BMC Pediatr 2018 Dec;18(1):232. doi: 10.1186/s12887-018-1206-8

10. Blomkvist EAM, Wills AK, Helland SH, Hillesund ER, Øverby NC. Effectiveness of a kindergarten-based intervention to increase vegetable intake and reduce food neophobia amongst 1-year-old children: a cluster randomised controlled trial. Food & Nutrition Research 2021 Oct 8;65. doi: 10.29219/fnr.v65.7679

11. Branco Dos Santos Lima R, Barbosa LP. Self-efficacy-based web and mobile intervention to improve infant sleep: Randomized trial with Brazilian mothers. Sleep Medicine 2025 Oct;134:106718. doi: 10.1016/j.sleep.2025.106718

12. Campbell KJ, Hesketh KD, McNaughton SA, Ball K, McCallum Z, Lynch J, Crawford DA. The extended Infant Feeding, Activity and Nutrition Trial (InFANT Extend) Program: a cluster-randomized controlled trial of an early intervention to prevent childhood obesity. BMC Public Health 2016 Dec;16(1):166. doi: 10.1186/s12889-016-2836-0

13. Chen Y, Wu Y, Dill S-E, Guo Y, Westgard CM, Medina A, Weber AM, Darmstadt GL, Zhou H, Rozelle S, Sylvia S. Effect of the mHealth-supported Healthy Future programme delivered by community health workers on maternal and child health in rural China: study protocol for a cluster randomised controlled trial. BMJ Open 2023 Jan;13(1):e065403. doi: 10.1136/bmjopen-2022-065403

14. Chen S, Ling J, Buhlman R, Tadavich S, Kao T-SA. Acceptability and satisfaction of a mindfulness-based healthy eating and stress management program targeting economically marginalized families in a pilot trial. Journal of Pediatric Psychology 2025 May 1;50(5):402–411. doi: 10.1093/jpepsy/jsaf010

15. Choonhawarakorn K, Kasemkhun P, Leelataweewud P. Effectiveness of a message service on child oral health practice via a social media application: A randomized controlled trial. Int J Paed Dentistry 2025 Mar;35(2):446–455. doi: 10.1111/ipd.13256

16. Delisle C, Sandin S, Forsum E, Henriksson H, Trolle-Lagerros Y, Larsson C, Maddison R, Ortega FB, Ruiz JR, Silfvernagel K, Timpka T, Löf M. A web- and mobile phone-based intervention to prevent obesity in 4-year-olds (MINISTOP): a population-based randomized controlled trial. BMC Public Health 2015 Dec;15(1):95. doi: 10.1186/s12889-015-1444-8

17. Dennis C-L, Marini F, Dick JA, Atkinson S, Barrett J, Bell R, Berard A, Berger H, Brown HK, Constantin E, Da Costa D, Feller A, Guttmann A, Janus M, Joseph KS, Jüni P, Kimmins S, Letourneau N, Li P, Lye S, Maguire JL, Matthews SG, Millar D, Misita D, Murphy K, Nuyt AM, O"Connor DL, Parekh RS, Paterson A, Puts M, Ray J, Roumeliotis P, Scherer S, Sellen D, Semenic S, Shah PS, Smith GN, Stremler R, Szatmari P, Telnner D, Thorpe K, Tremblay MS, Vigod S, Walker M, Birken C. Protocol for a randomised trial evaluating a preconception-early childhood telephone-based intervention with tailored e-health resources for women and their partners to optimise growth and development among children in Canada: a Healthy Life Trajectory Initiative (HeLTI Canada). BMJ Open 2021 Feb;11(2):e046311. doi: 10.1136/bmjopen-2020-046311

18. Downing KL, Salmon J, Hinkley T, Hnatiuk JA, Hesketh KD. A mobile technology intervention to reduce sedentary behaviour in 2- to 4-year-old children (Mini Movers): study protocol for a randomised controlled trial. Trials 2017 Dec;18(1):97. doi: 10.1186/s13063-017-1841-7

19. Downing KL, Salmon J, Hinkley T, Hnatiuk JA, Hesketh KD. Feasibility and Efficacy of a Parent-Focused, Text Message–Delivered Intervention to Reduce Sedentary Behavior in 2- to 4-Year-Old Children (Mini Movers): Pilot Randomized Controlled Trial. JMIR Mhealth Uhealth 2018 Feb 9;6(2):e39. doi: 10.2196/mhealth.8573

20. Downs SM, Sackey J, Kalaj J, Smith S, Fanzo J. An mHealth voice messaging intervention to improve infant and young child feeding practices in Senegal. Maternal & Child Nutrition 2019 Oct;15(4):e12825. doi: 10.1111/mcn.12825

21. Downs SM, Gueye D, Sall M, Ndoye B, Sarr NN, Sarr M, Mboup S, Alam NA, Diouf A, Merchant EV, Sackey J. The impact and implementation of an mHealth intervention to improve infant and young child feeding in Senegal: IIMAANJE protocol for a cluster randomized control trial. Front Public Health 2023 Sept 25;11:1258963. doi: 10.3389/fpubh.2023.1258963

22. Ferdous TE, Jaman MdJ, Siddique AB, Sultana N, Hossain T, Arifeen SE, Billah SM. Feasibility of Employing mHealth in Delivering Preventive Nutrition Interventions Targeting the First 1000 Days of Life: Experiences from a Community-Based Cluster Randomised Trial in Rural Bangladesh. Nutrients 2024 Oct 10;16(20):3429. doi: 10.3390/nu16203429

23. Garcia IL, Luoto J, Aboud F, Jervis P, Mwoma T, Alu E, Odhiambo A. In-person versus remote (mHealth) delivery for a responsive parenting intervention in rural Kenya: a cluster randomized controlled trial. BMC Public Health 2024 Sept 5;24(1):2421. doi: 10.1186/s12889-024-19828-5

24. Gago C, Aftosmes-Tobio A, Beckerman-Hsu JP, Oddleifson C, Garcia EA, Lansburg K, Figueroa R, Yu X, Kitos N, Torrico M, Leonard J, Jurkowski JK, Mattei J, Kenney EL, Haneuse S, Davison KK. Evaluation of a cluster-randomized controlled trial: Communities for Healthy Living, family-centered obesity prevention program for Head Start parents and children. Int J Behav Nutr Phys Act 2023 Jan 11;20(1):4. doi: 10.1186/s12966-022-01400-2

25. Ghofranipour F, Hamzavi Zarghani N, Mohammadi E, Mehrizi AAH, Tavousi M, De Craemer M, Cardon G. An internet-based educational intervention for mothers targeting preschoolers’ weight management promotion (PWMP): a pilot study. BMC Public Health 2022 Nov 29;22(1):2220. doi: 10.1186/s12889-022-14543-5

26. Hammersley ML, Jones RA, Okely AD. Time2bHealthy – An online childhood obesity prevention program for preschool-aged children: A randomised controlled trial protocol. Contemporary Clinical Trials 2017 Oct;61:73–80. doi: 10.1016/j.cct.2017.07.022

27. Hammersley ML, Okely AD, Batterham MJ, Jones RA. An Internet-Based Childhood Obesity Prevention Program (Time2bHealthy) for Parents of Preschool-Aged Children: Randomized Controlled Trial. J Med Internet Res 2019 Feb 8;21(2):e11964. doi: 10.2196/11964

28. He Q, Ha ASC, Zheng B, Okely AD. Feasibility and potential efficacy of a family‐based intervention on promoting physical activity levels and fundamental movement skills in preschoolers: A cluster randomised controlled trial. Applied Psych Health &amp; Well 2024 Aug;16(3):1266–1288. doi: 10.1111/aphw.12527

29. Heerman WJ, Perrin EM, Yin HS, Schildcrout JS, Delamater AM, Flower KB, Sanders L, Wood C, Kay MC, Adams LE, Rothman RL. The Greenlight Plus Trial: Comparative effectiveness of a health information technology intervention vs. health communication intervention in primary care offices to prevent childhood obesity. Contemporary Clinical Trials 2022 Dec;123:106987. doi: 10.1016/j.cct.2022.106987

30. Heerman WJ, Rothman RL, Sanders LM, Schildcrout JS, Flower KB, Delamater AM, Kay MC, Wood CT, Gross RS, Bian A, Adams LE, Sommer EC, Yin HS, Perrin EM, Greenlight Investigators, De La Barrera B, Bility M, Cruz Jimenez Smith M, Cruzatte EF, Guevara G, Howard JB, Lampkin J, Orr CJ, Pilotos McBride J, Quintana Forster L, Ramirez KS, Rodriguez J, Schilling S, Shepard WE, Soto A, Velazquez JJ, Wallace S. A Digital Health Behavior Intervention to Prevent Childhood Obesity: The Greenlight Plus Randomized Clinical Trial. JAMA 2024 Dec 24;332(24):2068. doi: 10.1001/jama.2024.22362

31. Helle C, Hillesund ER, Omholt ML, Øverby NC. Early food for future health: a randomized controlled trial evaluating the effect of an eHealth intervention aiming to promote healthy food habits from early childhood. BMC Public Health 2017 Dec;17(1):729. doi: 10.1186/s12889-017-4731-8

32. Helle C, Hillesund ER, Wills AK, Øverby NC. Evaluation of an eHealth intervention aiming to promote healthy food habits from infancy -the Norwegian randomized controlled trial Early Food for Future Health. Int J Behav Nutr Phys Act 2019 Dec;16(1):1. doi: 10.1186/s12966-018-0763-4

33. Helle C, Hillesund ER, Wills AK, Øverby NC. Examining the effects of an eHealth intervention from infant age 6 to 12 months on child eating behaviors and maternal feeding practices one year after cessation: The Norwegian randomized controlled trial Early Food for Future Health. Simeoni U, editor. PLoS ONE 2019 Aug 23;14(8):e0220437. doi: 10.1371/journal.pone.0220437

34. Henriksson H, Alexandrou C, Henriksson P, Henström M, Bendtsen M, Thomas K, Müssener U, Nilsen P, Löf M. MINISTOP 2.0: a smartphone app integrated in primary child health care to promote healthy diet and physical activity behaviours and prevent obesity in preschool-aged children: protocol for a hybrid design effectiveness-implementation study. BMC Public Health 2020 Dec;20(1):1756. doi: 10.1186/s12889-020-09808-w

35. Hesketh KD, Downing KL, Galland BC, Nicholson JM, Taylor R, Orellana L, Abdelrazek M, Koorts H, Brown V, Haines J, Campbell KJ, Barnett LM, Löf M, Moodie M, Carson V, Salmon J. Protocol for the Let’s Grow randomised controlled trial: examining efficacy, cost-effectiveness and scalability of a m-Health intervention for movement behaviours in toddlers. BMJ Open 2022 Mar;12(3):e057521. doi: 10.1136/bmjopen-2021-057521

36. Ihab M, El Din WE, Ammar N, Yassin R, El Tantawi M. Using mHealth to promote parents’ brushing of preschool children’s teeth: a protocol for a randomized factorial trial using the Multi-phase Optimization Strategy (MOST). Trials 2022 Dec;23(1):17. doi: 10.1186/s13063-021-05931-0

37. Ihab M, Abdelaziz WEE-D, Hassan W, El Tantawi M. Development and acceptability of behavioral interventions promoting mothers’ brushing of pre-school children’s teeth: The preparation phase of the multi-phase optimization strategy framework. BMC Oral Health 2023 Aug 31;23(1):616. doi: 10.1186/s12903-023-03351-x

38. Ihab M, El-Sherif Y, Yassin R, Nabil N, Quritum M, Balbaa N, Tantawi ME. Optimizing mHealth Interventions for Children’s Oral Hygiene: A Factorial Trial. J Dent Res 2024 Dec;104(2):155–163. doi: 10.1177/00220345241291985

39. Jiying L, Sisi C, Nanhua Z, Lorraine B. R, Jean M. K. Happy Family, Healthy Kids: A Healthy Eating and Stress Management Program in Low-Income Parent–Preschooler Dyads. Nursing Research 2024 Jan;73(1):3–15. doi: 10.1097/NNR.0000000000000697

40. Karssen LT, Vink JM, De Weerth C, Hermans RCJ, De Kort CPM, Kremers SP, Ruiter ELM, Larsen JK. An App-Based Parenting Program to Promote Healthy Energy Balance–Related Parenting Practices to Prevent Childhood Obesity: Protocol Using the Intervention Mapping Framework. JMIR Form Res 2021 May 14;5(5):e24802. doi: 10.2196/24802

41. Karssen LT, Larsen JK, Burk WJ, Kremers SPJ, Hermans RCJ, Ruiter ELM, Vink JM, De Weerth C. Process and effect evaluation of the app-based parenting program Samen Happie! on infant zBMI: A randomized controlled trial. Front Public Health 2022 Dec 23;10:1012431. doi: 10.3389/fpubh.2022.1012431

42. Kitsaras G, Allan J, Pretty IA. Bedtime Routines Intervention for Children (BRIC) using an automated text messaging system for behaviour change: study protocol for an early phase study. Pilot Feasibility Stud 2020 Dec;6(1):14. doi: 10.1186/s40814-020-0562-y

43. Lafave LMZ, Hayek J, Webster AD, McConnell C. Creating healthy eating and active environments in early learning settings: protocol of the CHEERS eHealth intervention study. Front Nutr 2024 Feb 28;11:1337873. doi: 10.3389/fnut.2024.1337873

44. Lee H, Oldewage-Theron W, Dawson J. Effects of a Theory-Based, Multicomponent eHealth Intervention for Obesity Prevention in Young Children from Low-Income Families: A Pilot Randomized Controlled Study. Nutrients 2023 May 13;15(10):2296. doi: 10.3390/nu15102296

45. Lewis KH, Hsu F-C, Block JP, Skelton JA, Schwartz MB, Krieger J, Hindel LR, Ospino Sanchez B, Zoellner J. A Technology-Driven, Healthcare-Based Intervention to Improve Family Beverage Choices: Results from a Pilot Randomized Trial in the United States. Nutrients 2023 Apr 29;15(9):2141. doi: 10.3390/nu15092141

46. Ling J, Suriyawong W, Robbins LB, Zhang N, Kerver JM. FirstStep2Health : A cluster randomised trial to promote healthy behaviours and prevent obesity amongst low‐income preschoolers. Pediatric Obesity 2024 July;19(7):e13122. doi: 10.1111/ijpo.13122

47. Ling J, Ashley A, Zahry N, Kao T-SA, Wahman CL, Resnicow K, Robbins LB, Kerver JM, Zhang N. A Mindfulness-Based Lifestyle Intervention Among Economically Marginalized Caregiver-Preschooler Dyads: Feasibility, Acceptability, and Satisfaction. School Mental Health 2025 June;17(2):715–732. doi: 10.1007/s12310-025-09767-w

48. Lotto M, Strieder AP, Ayala Aguirre PE, Oliveira TM, Andrade Moreira Machado MA, Rios D, Cruvinel T. Parental-oriented educational mobile messages to aid in the control of early childhood caries in low socioeconomic children: A randomized controlled trial. Journal of Dentistry 2020 Oct;101:103456. doi: 10.1016/j.jdent.2020.103456

49. Lozada-Tequeanes AL, Théodore FL, Kim-Herrera E, García-Guerra A, Quezada-Sánchez AD, Alvarado-Casas R, Bonvecchio A. Effectiveness and Implementation of a Text Messaging mHealth Intervention to Prevent Childhood Obesity in Mexico in the COVID-19 Context: Mixed Methods Study. JMIR Mhealth Uhealth 2024 Apr 9;12:e55509. doi: 10.2196/55509

50. Markides BR, Hesketh KD, Maddison R, Laws R, Denney-Wilson E, Campbell KJ. Fussy Eating Rescue, a mobile-web app for responsive feeding practises among parents of toddlers: protocol for a pilot randomised controlled feasibility trial. Pilot Feasibility Stud 2023 July 22;9(1):128. doi: 10.1186/s40814-023-01278-2

51. Militello L, Melnyk BM, Hekler EB, Small L, Jacobson D. Automated Behavioral Text Messaging and Face-to-Face Intervention for Parents of Overweight or Obese Preschool Children: Results From a Pilot Study. JMIR mHealth uHealth 2016 Mar 14;4(1):e21. doi: 10.2196/mhealth.4398

52. Nezami BT, Lytle LA, Tate DF. A randomized trial to reduce sugar-sweetened beverage and juice intake in preschool-aged children: description of the Smart Moms intervention trial. BMC Public Health 2016 Dec;16(1):837. doi: 10.1186/s12889-016-3533-8

53. Nezami BT, Ward DS, Lytle LA, Ennett ST, Tate DF. A mHealth randomized controlled trial to reduce sugar‐sweetened beverage intake in preschool‐aged children. Pediatric Obesity 2017 Nov;13(11):668–676. doi: 10.1111/ijpo.12258

54. Nyström CD, Sandin S, Henriksson P, Henriksson H, Trolle-Lagerros Y, Larsson C, Maddison R, Ortega FB, Pomeroy J, Ruiz JR, Silfvernagel K, Timpka T, Löf M. Mobile-based intervention intended to stop obesity in preschool-aged children: the MINISTOP randomized controlled trial ,. The American Journal of Clinical Nutrition 2017 June;105(6):1327–1335. doi: 10.3945/ajcn.116.150995

55. Nyström CD, Sandin S, Henriksson P, Henriksson H, Maddison R, Löf M. A 12-month follow-up of a mobile-based (mHealth) obesity prevention intervention in pre-school children: the MINISTOP randomized controlled trial. BMC Public Health 2018 Dec;18(1):658. doi: 10.1186/s12889-018-5569-4

56. Øverby NC, Hillesund ER, Helland SH, Helle C, Wills AK, Lamu AN, Osorio NG, Lian H, Ersfjord TI, Van Daele W, Bjørkkjær T, Valen EN, Gebremariam MK, Grasaas E, Kiland C, Schwarz UVT, Abel MH, Love P, Campbell K, Rutter H, Barker ME, Vik FN, Medin AC. Evaluating the effectiveness and implementation of evidence-based early-life nutrition interventions in a community setting a hybrid type 1 non-randomized trial – the Nutrition Now project protocol. Front Endocrinol 2023 Jan 10;13:1071489. doi: 10.3389/fendo.2022.1071489

57. Pakarinen A, Flemmich M, Parisod H, Selänne L, Hamari L, Aromaa M, Leppänen V, Löyttyniemi E, Smed J, Salanterä S. Protocol for digital intervention for effective health promotion of small children—A cluster randomized trial. Journal of Advanced Nursing 2018 July;74(7):1685–1699. doi: 10.1111/jan.13561

58. Peden M, Eady M, Okely A, Patterson K, Batterham M, Jones R. A blended professional learning intervention for early childhood educators to target the promotion of physical activity and healthy eating: the HOPPEL cluster randomized stepped-wedge trial. BMC Public Health 2022 Dec;22(1):1353. doi: 10.1186/s12889-022-13542-w

59. Po’e EK, Heerman WJ, Mistry RS, Barkin SL. Growing Right Onto Wellness (GROW): A family-centered, community-based obesity prevention randomized controlled trial for preschool child–parent pairs. Contemporary Clinical Trials 2013 Nov;36(2):436–449. doi: 10.1016/j.cct.2013.08.013

60. Raat H, Struijk MK, Remmers T, Vlasblom E, Van Grieken A, Broeren SM, Te Velde SJ, Beltman M, Boere-Boonekamp MM, L’Hoir MP. Primary prevention of overweight in preschool children, the BeeBOFT study (breastfeeding, breakfast daily, outside playing, few sweet drinks, less TV viewing): design of a cluster randomized controlled trial. BMC Public Health 2013 Dec;13(1):974. doi: 10.1186/1471-2458-13-974

61. Ramírez AS, Ayala GX, Murillo M, Glik DC, Guerrero AD. Integrating Theory With a User-Centered Design Approach to Maximize mHealth Acceptability and Usability. Health Educ Behav 2025 June;52(3):329–339. doi: 10.1177/10901981241311232

62. Riera-Navarro C, Schwartz C, Ducrot P, Noirot L, Delamaire C, Sales-Wuillemin E, Semama DS, Lioret S, Nicklaus S. A web-based and mobile randomised controlled trial providing complementary feeding guidelines to first-time parents in France to promote responsive parental feeding practices, healthy children’s eating behaviour and optimal body mass index: the NutrienT trial study protocol. BMC Public Health 2024 Sept 27;24(1):2649. doi: 10.1186/s12889-024-20057-z

63. Røed M, Hillesund ER, Vik FN, Van Lippevelde W, Øverby NC. The Food4toddlers study - study protocol for a web-based intervention to promote healthy diets for toddlers: a randomized controlled trial. BMC Public Health 2019 Dec;19(1):563. doi: 10.1186/s12889-019-6915-x

64. Røed M, Medin AC, Vik FN, Hillesund ER, Van Lippevelde W, Campbell K, Øverby NC. Effect of a Parent-Focused eHealth Intervention on Children’s Fruit, Vegetable, and Discretionary Food Intake (Food4toddlers): Randomized Controlled Trial. J Med Internet Res 2021 Feb 16;23(2):e18311. doi: 10.2196/18311

65. Sandborg J, Downing KL, Orellana L, Taylor RW, Barnett LM, Carson V, Hesketh KD. Six-month intervention effect of a digital movement behavior intervention on parent- and child intermediary outcomes—results from the Let’s Grow randomized controlled trial. Int J Behav Nutr Phys Act 2025 June 16;22(1):78. doi: 10.1186/s12966-025-01764-1

66. Seyyedi N, Rahimi B, Eslamlou HRF, Afshar HL, Spreco A, Timpka T. Smartphone-Based Maternal Education for the Complementary Feeding of Undernourished Children Under 3 Years of Age in Food-Secure Communities: Randomised Controlled Trial in Urmia, Iran. Nutrients 2020 Feb 24;12(2):587. doi: 10.3390/nu12020587

67. Thomas K, Löf M, Lundgren M, Fagerström M, Hesketh KD, Brown V, Häbel H, Delisle Nyström C. MINISTOP 3.0: Implementation of a mHealth obesity prevention program within Swedish child healthcare – study protocol for a cluster randomized controlled trial. BMC Public Health 2024 Sept 27;24(1):2594. doi: 10.1186/s12889-024-20137-0

68. Tomayko EJ, Prince RJ, Cronin KA, Parker T, Kim K, Grant VM, Sheche JN, Adams AK. Healthy Children, Strong Families 2: A randomized controlled trial of a healthy lifestyle intervention for American Indian families designed using community-based approaches. Clinical Trials 2017 Apr;14(2):152–161. doi: 10.1177/1740774516685699

69. Tomayko EJ, Prince RJ, Cronin KA, Kim K, Parker T, Adams AK. The Healthy Children, Strong Families 2 (HCSF2) Randomized Controlled Trial Improved Healthy Behaviors in American Indian Families with Young Children. Current Developments in Nutrition 2019 Aug;3:53–62. doi: 10.1093/cdn/nzy087

70. Van Grieken A, Vlasblom E, Wang L, Beltman M, Boere-Boonekamp MM, L’Hoir MP, Raat H. Personalized Web-Based Advice in Combination With Well-Child Visits to Prevent Overweight in Young Children: Cluster Randomized Controlled Trial. J Med Internet Res 2017 July 27;19(7):e268. doi: 10.2196/jmir.7115

71. Wang K, Lee GHM, Liu P, Gao X, Wong SYS, Wong MCM. Health belief model for empowering parental toothbrushing and sugar intake control in reducing early childhood caries among young children—study protocol for a cluster randomized controlled trial. Trials 2022 Dec;23(1):298. doi: 10.1186/s13063-022-06208-w

72. Willis EA, Burney R, Hales D, Ilugbusi LO, Tate DF, Nezami B, Clarke EC, Moore RH, Mathews E, Thompson M, Beckelheimer B, Ward DS. “My wellbeing-their wellbeing “– An eHealth intervention for managing obesity in early care and education: Protocol for the Go NAPSACC Cares cluster randomized control trial. Shen L, editor. PLoS ONE 2023 July 7;18(7):e0286912. doi: 10.1371/journal.pone.0286912

73. Wu Q, Wang X, Zhang J, Zhang Y, Van Velthoven MH. The effectiveness of a WeChat-based self-assessment with a tailored feedback report on improving complementary feeding and movement behaviour of children aged 6–20 months in rural China: a cluster randomized controlled trial. The Lancet Regional Health - Western Pacific 2023 Aug;37:100796. doi: 10.1016/j.lanwpc.2023.100796

74. Yoong SL, Lum M, Leung GKW, Pearson N, Truby H, Dix C, Moumin NA, Wolfenden L, Ananthapavan J, Grady A, Wiggers J, Delaney T, Rychetnik L, Romiti M, Lamont H, Stanley S, Lim M, Oldmeadow C, Mastersson N, Sutherland R. Tiny Bites, a digital health intervention delivered in early childhood education and care centres to support educators and caregivers to prevent childhood obesity: study protocol for a cluster randomised controlled trial. BMJ Open 2025 Nov;15(11):e106436. doi: 10.1136/bmjopen-2025-106436

75. Yoshizaki A, Mohri I, Yamamoto T, Shirota A, Okada S, Murata E, Hoshino K, Kato-Nishimura K, Matsuzawa S, Kato T, Taniike M. An Interactive Smartphone App, Nenne Navi, for Improving Children’s Sleep: Pilot Usability Study. JMIR Pediatr Parent 2020 Dec 1;3(2):e22102. doi: 10.2196/22102

76. Yoshizaki A, Murata E, Yamamoto T, Fujisawa TX, Hanaie R, Hirata I, Matsumoto S, Mohri I, Taniike M. Improving Children’s Sleep Habits Using an Interactive Smartphone App: Community-Based Intervention Study. JMIR Mhealth Uhealth 2023 Feb 10;11:e40836. doi: 10.2196/40836

77. Zhang Q, Panichelli J, Hall LA. Assessment of Cooking Matters Facebook Platform to Promote Healthy Eating Behaviors among Low-Income Caregivers of Young Children in the United States: A Pilot Study. Nutrients 2021 Aug 4;13(8):2694. doi: 10.3390/nu13082694
