# Supplementary material for: Digital and Blended Lifestyle Interventions for Preschool-Aged Children and Families With a Low Socioeconomic Position and the General Population: Scoping Review
Source: J Med Internet Res. 2026 Jun 5;28:e86596. doi: 10.2196/86596 (PMC13240985; doi:10.2196/86596)
Supplement: Multimedia Appendix 5 [file jmir-v28-e86596-s005.docx]

| **Intervention name and goal** | **Target behaviours** | **Target group** | **Theories, frameworks, & evidence-based guidelines** | **Strategy, Channel** | **Delivery environment** | **PSD features** | **Effectiveness** |
| --- | --- | --- | --- | --- | --- | --- | --- |
| **General population** |  |  |  |  |  |  |  |
| barnE-mat  *Promote early healthy, sustainable diet & healthy food habits in children by encouraging beneficial parental feeding practices, enhancing parental self-efficacy, skills, & confidence with respect to child feeding.* | Nutrition | Parents/Caregivers | Attachment-theory  Social Cognitive Theory  Framework of anticipatory guidance | Digital  Website | Daily life | *Suggestions* (tips, recipes, tutorials)  *Tailoring* (monthly updating & age-adjusted website)  *Reduction* (information according to age of children) | More effective (ineffective at 12 months follow-up) |
| Barns matmot 2.0  *Promote a healthy and varied diet amongst 1-year-old children*. | Nutrition | Kindergarten staff  Parents/Caregivers | Nothing reported | Blended  Web-page  In-person implementation through kindergarten staff (e.g. warm meals) | Daily life and kindergartens | Not identifiable | Less effective |
| Bedtime Routines Intervention for Children (BRIC)  *Support the adoption and maintenance of optimal bed time routines for first time parents that will lead to measurable improvements in health, development and wellbeing for young children* | Sleep | Parents/Caregivers | Behaviour Change Wheel | Digital  Text-messages | Daily life | Not identifiable | N/A |
| Billah et al.  *Improve child feeding practices* | Nutrition | Parents/Caregivers with professional intermediate | Evidence-based recommendations, guidelines, & previous research | Blended  In-person sessions  Software application (to aid professionals in counselling) | Daily life and clinic | Not identifiable | More effective |
| Branco dos Santos Lima & Pinheiro Barbosa  *Reducing nighttime awakenings and increasing total sleep time in infants* | Sleep | Parents/Caregivers | Evidence-based guidelines, & previous research  Self-efficacy Theory | Blended  Online meetings (with researcher)  Text-messages  Online diary | Daily life | *Reminders* (via Whatsapp)  *Self-monitoring* (online diary) | More effective |
| CHEERS  *Improving the knowledge, beliefs and adherence to best practices of early childhood educators* | Nutrition  Physical activity | Early childhood educators | Social Cognitive Theory | Digital  Online modules | ECEC centers | *Reminders* (email) | N/A |
| Choonhawarakorn, Kasemkhun, & Leelataweewud  *Promotion of healthy oral health behaviours that contribute to ECC prevention* | Nutrition  Oral health | Parents/Caregivers | Protection motivation theory | Digital  Text-messages | Daily life | *Tailoring* (information tailored to age and development) | Less effective |
| E-health4Uth Healthy toddler  *Provides tailored health education for parents regarding key behaviours related to childhood overweight prevention* | Nutrition  Physical activity  Sedentary behaviour | Parents/Caregivers | Social-ecological model  Theory of Planned Behaviour  Social Cognitive Theory | Blended  Internet-based E-health module  Email  Youth Health Care (YHC) counselling sessions (in-person) | Daily life and YHC organization centers | *Tailoring* (tailored information & advice)  *Suggestions* (advice on behaviour change)  *Reminder* (email) | Ineffective |
| Food4toddlers  *Influence child diet quality & food variety by targeting main parents/caregivers & their awareness of the food and eating environments* | Nutrition | Parents/Caregivers | Model of Planned Promotion for Population Health  Theory of Planned Behaviour  Social Cognitive Theory  BCTs (unspecified framework)  Evidence-based recommendations, guidelines, & previous research | Digital  Website | Daily life | *Suggestions* (suggest healthy behaviours & recipes)  *Reduction* (topics into different modules & chapters)  *Tunneling* (not all content visible, just first 2 chapters)  *Verifiability* (links to recommended websites)  *Social learning* (discussion forum)  *Similarity* (messages & video actors of same age used) | Less effective |
| Fussy Eating Rescue  *Improve the feeding practices of parents of toddlers concerned about fussy eating* | Nutrition | Parents/Caregivers | Theoretical Domains framework  IDEAS (Integrate, Design, Assess, and Share) Framework  Behaviour Change Wheel  Behaviour Change Taxonomy v1  Evidence-based recommendations, guidelines, & previous research | Digital  Text-messages  Web-based app | Daily life | *Personalization* (messages based on child’s age, current practises, goals, & preferences)  *Suggestions* (SMS with tips & recipes)  *Reminders* (prompts to reengage)  *Self-monitoring* (tracker of food and child's responses) | N/A |
| Go NAPSACC  *Increase use of healthy eating and physical activity evidence-based practices* | Nutrition  Physical activity | ECE teachers  Children | Social Cognitive Theory  Social Ecological Model  BCTs (unspecified) | Blended  Materials (microfiber towel, water bottle, exercise bands, Fitbit, Aria Air smart scale)  Mobile web-app  Text-messages | Daily life and pre-schools | *Self-monitoring* (Fitbit)  *Tailoring* (tailored feedback)  *Personalization* (teacher receive daily food goal)  *Reminders* (to track)  *Suggestions* (general tips)  *Tunneling* (users guided through new lessons) | N/A |
| Greenlight Plus  *Childhood obesity prevention* | Nutrition  Physical activity  Sleep | Parents/Caregivers | Social Cognitive Theory  Active Learning  Evidence-based recommendations, guidelines, & previous research | Digital  Text-messages  Web-based “dashboard” | Daily life | *Self-monitoring* (health behaviour goals)  *Personalization* (“dashboard”)  *Reduction* (8 modules with developmentally appropriate behavioural goals)  *Suggestions* (progress on goals with behaviour tips) | More effective |
| He et al.  *Support their children's physical activity and fundamental movement skills* | Physical activity | Parents/Caregivers | BCTs (Michie et al.) | Blended  In-person workshop session  Materials (Logbook)  Social media (Whatsapp group) | Daily life and unspecified (location of in-person workshop unclear) | *Social learning* (Whatsapp group) | More effective |
| Healthy Life Trajectory Initiative (HeLTI) Canada  *Reduce the rate of child overweight and obesity* | Nutrition  Physical activity  Sedentary behaviour  Sleep | Parents/Caregivers | Nothing reported | Blended  Telephone-based collaborative care (sessions)  Web-based app | Daily life | *Personalization* (health information in app)  *Expertise* (expert recommended resources) | N/A |
| HOPPEL (Healthy Online Professional Program for Early Learners)  *Promote physical activity and healthy eating environments and policies and the physical activity levels of children in ECEC services* | Physical activity  Nutrition  Sedentary behaviour | Early childhood educators | Guskey’s model of teacher change  Community of Practice | Blended  In-person six-hour professional learning workshop  Online elements (asynchronous weekly blog, asynchronous forums) | Daily life and unspecified (location of face-to-face sessions unclear) | *Social learning* (asynchronous forums) | More effective |
| Ihab et al.  *Promote parental brushing of children’s teeth* | Oral health | Parents/Caregivers | Multi-phase Optimization Strategy (MOST) framework  Theory of Planned Behaviour | Blended  Motivational Interviewing sessions (in-person)  Materials (pamphlets)  Text-messages | Daily life | *Personalization* (messages: “{name}, this is {dentist} from {clinic})  *Expertise* (featuring a dentist) | Ineffective |
| InFANT Extend Program  *Early childhood obesity prevention* | Nutrition  Physical activity  Sedentary behaviour | Parents/Caregivers | Nothing reported | Blended  Web-pages  Social media (Facebook group)  Intervention sessions (in-person) | Daily life and unspecified (location of intervention sessions not clear) | *Reminders* (monthly emails and FB posts)  *Social learning* (Facebook group) | N/A |
| Let's Grow  *Improve the composition of movement behaviours in children from 2 years* | Physical activity  Sleep  Sedentary behaviour | Parents/Caregivers | Michie's Behaviour Change Wheel (BCTs)  CALO-RE taxonomy (BCTs)  Social Cognitive Theory – Family Perspective  Family Ecological Model  Chaos theory  Complex dynamic systems | Digital  Web-based app  Text-messages | Daily life | *Tunneling* (open new module after completion)  *Reminders* (via SMS)  *Personalization* (SMS include parent and child names)  *Social learning* (social forum) | Less effective |
| MINISTOP  *Have the pre-school children achieve healthy body fatness through increasing physical activity and improving dietary behaviour* | Nutrition  Physical activity  Sedentary behaviour | Parents/Caregivers | Social Cognitive Theory  CALO-RE Taxonomy (BCTs)  Evidence-based recommendations, guidelines, & previous research | Digital  Smartphone application | Daily life | *Reminders* (emails)  *Expertise* (dietitian as part of Facebook group)  *Suggestions* (advice)  *Social learning* (Facebook group) | Less effective  (Ineffective at 12-month follow-up) |
| MINISTOP 2.0  *Improving dietary- and physical activity behaviours & preventing prevalence of overweight and obesity in preschool-aged children* | Nutrition  Physical activity  Sedentary behaviour  Sleep  Oral health | Parents/Caregivers | Social Cognitive Theory  Behaviour Change Taxonomy v1  Evidence-based recommendations, guidelines, & previous research | Digital  Smartphone app | Daily life | *Suggestions* (push notifications & library module)  *Reminders* (push notifications)  *Self-monitoring* (self-monitoring module)  *Personalization* (feedback based on entries)  *Reduction* (modules with different subjects)  *Tunneling* (change in themes guided by system)  *Rewards* (medals) | More effective |
| MINISTOP 3.0  *To improve lifestyle behaviours in two- to three-year-old children* | Nutrition  Physical activity  Sedentary behaviours | Parents/Caregivers | Evidence-based recommendations, guidelines, & previous research  Social Cognitive Theory  Behaviour Change Taxonomy v1 | Digital  Smartphone app | Daily life | *Suggestions* (through library)  *Expertise* (films of dietician answering commonly asked questions)  *Self-monitoring* (can register child's intake, physical activity and screen time) | N/A |
| Mini Movers  *Help parents of children aged 4 years to achieve a healthy weight and body fat* | Sedentary behaviour | Parents/Caregivers | Evidence-based recommendations, guidelines, & previous research  CALO-RE taxonomy (BCTs)  Social Cognitive Theory | Blended  Materials  In-person discussion session  Text-messages | Daily life | *Personalization* (text messages)  *Suggestions* (advice & strategies)  *Verifiability* (links to reputable website) | More effective |
| Nenne Navi  *To positively influence caregivers’ behaviour to ensure healthy sleep habits among young children* | Sleep | Parents/Caregivers | Behavioural therapy | Digital  Smartphone app | Daily life | *Suggestions* (sends advice and suggestions)  *Personalization* (personalized advice)  *Self-monitoring* (e.g. night awakenings, morning mood, & time children fall asleep) | More effective |
| NutrienT  *Promoting the establishment of healthy eating habits and preventing childhood obesity* | Nutrition | Parents/Caregivers | BCT taxonomy (Michie et al.) | Blended  Smartphone app  Materials (brochure) | Daily life | *Trustworthiness* (messages from credible sources)  *Reminders* | N/A |
| Nutrition now resource  *Support healthy dietary behaviours* | Nutrition | Parents/Caregivers | Dynamic Integrated Evaluation Model (DIEM) | Digital  Website | Family-, MCH centre- and ECEC- settings | *Tailoring* (content and structure adapted to user context and needs) | N/A |
| Parents in Child Nutrition Informing Community (PICNIC)  *Engaging parents of young children & influencing social norms around nutrition & child feeding practices* | Nutrition | Parents/Caregivers | Theory of Planned Behaviour  Evidence-based recommendations, guidelines, & previous research | Blended  In-person peer education  Website  Social media (Facebook group) | Daily life and unspecified (location of peer education not clear) | *Reduction* (information organized by age group)  *Reminders* (via Facebook group)  *Similarity* (messages in "meme style")  *Social learning* (Facebook group) | N/A |
| Preschooler's weight management promotion, (PWMP)  *Promote weight management in preschool children of Tehran with any weight status* | Nutrition  Physical activity | Parents/Caregivers | Precede-Procede Model  Social Cognitive Theory | Digital  Text-messages | Daily life | *Reduction* (content per week) | More effective |
| Ready, Set, Gulp!  *Reduce sugar-sweetened beverage (SSB) and fruit juice (FJ) consumption in parent–child dyads* | Nutrition | Family | The Revised Family Ecological Model  Evidence-based recommendations, guidelines, & previous research | Blended  Materials (water promotion toolkit)  Smartphone app | Daily life | *Self-monitoring* (goal setting & tracking of beverage intake) | Less effective |
| Smart Moms  *Target maternal dietary behaviours that to promote positive changes child's dietary intake* | Nutrition | Parents/Caregivers | Social Cognitive Theory  BCTs (unspecified framework) | Blended  In-person group session  Materials (paper diary, printed list, charts, stickers, token prizes)  Text-messages | Daily life | *Personalization* (feedback in emails)  *Suggestions* (tips via text-messages)  *Reminders* (text-message with link to lesson) | More effective |
| TEXT2COPE  *Support healthy lifestyle behaviours of parents of overweight and obese preschoolers* | Nutrition  Physical activity | Parents/Caregivers | CBT (guiding principles)  Fogg's Behaviour Model  Evidence-based recommendations, guidelines, & previous research  Cognitive Theory  SMART goals framework | Blended  Face-to-face clinic visits/sessions  Text-messages | Daily life and primary care clinic | *Reminders*  *Praise* (message reinforcing positive behaviours)  *Personalization* (custom text message) | More effective |
| Time2bHealthy  *Facilitating behaviour change in preschool-aged children who are overweight or at risk of becoming overweight* | Nutrition  Physical activity  Sedentary behaviour  Sleep | Parents/Caregivers | Social Cognitive Theory  SMART goal framework  Evidence-based recommendations, guidelines, & previous research  Intervention Mapping | Digital  Website (with modules)  Weekly reminder emails  Social media (Facebook group) | Daily life | *Reminders* (emails)  *Expertise* (Dietitian as part of Facebook group)  *Social learning* (Facebook group) | Ineffective |
| TinyBites  *Target extending breastfeeding duration, infant feeding and responsive feeding practices, and child dietary intake* | Nutrition | Parents/Caregivers  ECEC services (receive implementation support) | Evidence-based recommendations & previous research  COM-B Model  BCTs (unspecified framework) | Blended  Text-messages  E-newsletters  Access to webinars  Web resources (digital toolbox)  Educational outreach visits (for ECEC staff) | Daily life | *Tailoring* (text-messages tailored to child’s age) | N/A |
| Wang et al.  *Promoting oral health of young children by reducing dental caries* | Oral health  Nutrition | Parents/Caregivers | Health Belief Model | Digital  Text-messages | Daily life | Not identifiable | N/A |
| WellWe  *Supporting the participation of families with small children in the promotion of their own health* | Physical activity  Nutrition | Parents/Caregivers | Mediating variable model  Family-centred approach  Mindfulness  Medical Research Council's framework for developing and evaluating complex interventions  Self-efficacy Theory  Evidence-based recommendations, guidelines, & previous research | Digital  Web-based app | Daily life | Not identifiable | N/A |
| Wu et al.  *Promote appropriate complementary feeding and movement behaviours* | Nutrition  Physical activity | Parents/Caregivers | Evidence-based recommendations, guidelines, & previous research | Digital  Social media app | Daily life | *Tailoring* (tailored feedback report based on answers & recommendations)  *Suggestions* (corresponding recommendations)  *Social learning* (WeChat is a social media platform) | More effective |
|  |  |  |  |  |  |  |  |
| **Low SEP** |  |  |  |  |  |  |  |
| Communities for Healthy Living (CHL)  *Obesity prevention* | Nutrition  Physical activity  Sleep  Sedentary behaviour | Parents/Caregivers | Community-based participatory research approach | Blended  Education sessions (in-person)  Social media (Facebook group)  Materials (letter, brochures, posters, flyers)  Online components (online Neighbour Resource Map) | Daily life and unspecified (location of education sessions unclear) | *Social learning* (Facebook group) | More effective |
| Cooking Matters  *Provide low-income caregivers with essential skills in shopping for and cooking healthy meals* | Nutrition | Parents/Caregivers | Not reported | Digital  Social media (Facebook group) | Daily life | *Social learning* (Facebook group) | Less effective |
| Downs et al.  *Improve infant and young child feeding (IYCF) practices in rural Senegal* | Nutrition | Parents/Caregivers | Theory of Planned Behaviour  Evidence-based recommendations, guidelines, & previous research | Digital  Voice messages | Daily life | *Suggestions* (messages provide advice on behaviour change) | Less effective |
| eHEROs  *Promoting healthy eating and activity behaviours and environments and, ultimately, healthy growth* | Nutrition  Physical activity | Parents/Caregivers | Social Cognitive Theory | Digital  Web-based smartphone app | Daily life | Not identifiable | N/A |
| FirstStep2Health  *Improving healthy lifestyle behaviours and preventing obesity amongst low-income preschoolers* | Nutrition  Physical activity  Sedentary behaviour | Parents/Caregivers  Children | Actor-Partner Interdependence Model (APIM) | Blended  Social media (Facebook-based programme)  Virtual meetings  Text-messages  Materials (flyers, cookbook, plates, community resource booklet, child letters to parents)  Daycare-based child programme | Daily life  Daycare | *Social learning* (Facebook programme) | More effective |
| FUNS  *Promote healthy weight of preschool-aged children* | Nutrition  Physical activity  Sedentary behaviour | Multiple caregivers (moms, dads, grandparents) | Family Interaction Theory  BCTs (unspecified framework) | Digital  Smartphone app | Daily life | *Social learning* (app includes online community)  *Reminders* (to practice)  *Personalization* (preferred time for the delivery of daily text messages)  *Self-monitoring* (individual-level tracking)  *Tunneling* (provides step-by-step guidance to navigate system)  *Praise* (‘Great job!’ after answering a question) | N/A |
| Futuros Fuertes  *Promote healthy feeding, screen time, and sleep practices among low-income Latino infants* | Nutrition  Sedentary behaviour  Sleep | Parents/Caregivers | Information-Motivation-Behavioural Skills (IMB) model of health behaviour change | Blended  Health education Sessions (in-person)  Text-messages | Daily life and primary care clinic | *Reminders*  *Similarity* (emojis)  *Reduction* (reducing content to specific ages) | Less effective |
| Growing Right Onto Wellness (GROW)  *Prevent childhood obesity* | Nutrition  Physical activity | Parents/Caregivers | Centers for Disease Control and Prevention's theory  Social Cognitive Theory  BCT taxonomy (Michie et al.) | Blended  In-person sessions  Social media | Daily life and community recreation center | *Social learning* (social media) | N/A |
| Happy Family, Healthy Kids  *Focused on healthy eating and stress management* | Nutrition | Parents/Caregivers  Children | Social Cognitive Theory | Blended  Materials (Crockpot, cookbook, plate, community resource booklet)  Online videos  Text-messages  In-person healthy diet participatory learning sessions for children specifically | Daily life  Daycare centers  Head Start centres | *Tunneling* (content with weekly videos) | Less effective |
| Healthy Children, Strong Families 2  *Mitigate obesity risk in urban and rural American Indian families* | Nutrition  Physical activity  Sedentary behaviour | Parents/Caregivers | Not reported | Blended  Materials (Toolkit (books & incentives))  Text-messages  Community-specific Facebook pages | Daily life | *Social learning* (Facebook pages)  *Suggestions* (posts including e.g. healthy recipe ideas, sleep tips) | Less effective |
| Healthy Future Programme  *Improve child health and maternal well-being* | Nutrition | Parents/Caregivers with professional intermediate | Theory of change  Evidence-based recommendations, guidelines, & previous research | Blended  App  In-person visits/sessions | Daily life | Not identifiable | N/A |
| IIMAANJE  *Aimed to improve infant and young child feeding (IYCF) practices* | Nutrition | Parents/Caregivers | Theory of Planned Behaviour  Positive Deviance  Evidence-based recommendations, guidelines, & previous research | Digital  Text-messages | Daily life | Not identifiable | N/A |
| Lee, Oldewage-Theron, & Dawson  *Improving child health behaviours and parental psychosocial attributes and feeding practices* | Nutrition  Physical activity | Parents/Caregivers | Social Cognitive Theory  SMART goals framework | Digital  Newsletters  Text-messages  Website | Daily life | *Reminders* (text-messages)  *Self-monitoring* (track SMART goals)  *Suggestions* (received online cooking tutorials) | Less effective |
| Ling et al.  *Target the connections among physical activity, dietary intake, and mental health to improve both preschoolers’ and caregivers’ adaptive coping, such as mindful eating, movement, and parenting to enhance their overall health* | Physical activity  Nutrition | Parents/Caregivers  Children  Facilitated through childcare teachers | Actor-Partner Interdependence Model  Allostatic Load Model  Transactional Theory of Stress and Coping | Blended  Classes in childcare  Caregiver group meetings (via Zoom)  Text-messages  Social media (Facebook group)  Website | Daily life  Childcare centers | *Reminders* (via website or Facebook)  *Social facilitation* (Facebook group) | N/A |
| Lotto et al.  *Aid in the control of early childhood caries (ECC) in low socioeconomic children* | Nutrition  Oral health | Parents/Caregivers | Evidence-based recommendations, guidelines, & previous research  Health Belief Model | Digital  Text-messages | Daily life | Not identifiable | Less effective |
| Msingi Bora  *Support early childhood development and responsive parenting* | Nutrition | Parents/Caregivers | Nothing reported | Blended  Delivery via smartphone (e.g. access to video content)  Social media (Whatsapp group)  In-person sessions | Daily life and unspecified (location of sessions unclear) | *Social learning* (Whatsapp group)  *Expertise* (expert as part of Whatsapp group) | N/A |
| NUTRES  *Prevent childhood overweight/obesity* | Nutrition  Physical activity | Parents/Caregivers | Evidence-based recommendations, guidelines, & previous research  Theory of Planned Behaviour | Digital  Text-messages | Daily life | *Suggestions* (dissemination of tips and recipes)  *Personalization* (text-messages to each participant) | Less effective |
| Samen Happie!  *Stimulate healthy child weight development among low SES families, by encouraging healthy energy balance-related parenting practices* | Nutrition  Sleep  Sedentary behaviour  Physical activity | Parents/Caregivers | Intervention Mapping Protocol  Evidence-based recommendations, guidelines, & previous research  BCTs (Michie et al.; Kok et al.) | Digital  App | Daily life | *Tailoring* (program content)  *Personalization* (use name, sex, birth date of child to personalize texts)  *Reduction* (age-based modules)  *Reminders* (reminders for goal and action plan)  *Self-monitoring* (personal goal or action plan) | Less effective |
| Seyyedi et al.  *Prevent child undernourishment by providing mothers with nutritional education* | Nutrition | Parents/Caregivers | Evidence-based recommendations, guidelines, & previous research | Digital  App | Daily life | *Expertise* (a chat function with clinicians) | More effective |
