# Supplementary material for: Digital and Blended Lifestyle Interventions for Preschool-Aged Children and Families With a Low Socioeconomic Position and the General Population: Scoping Review
Source: J Med Internet Res. 2026 Jun 5;28:e86596. doi: 10.2196/86596 (PMC13240985; doi:10.2196/86596)
Supplement: Multimedia Appendix 6 [file jmir-v28-e86596-s006.docx]

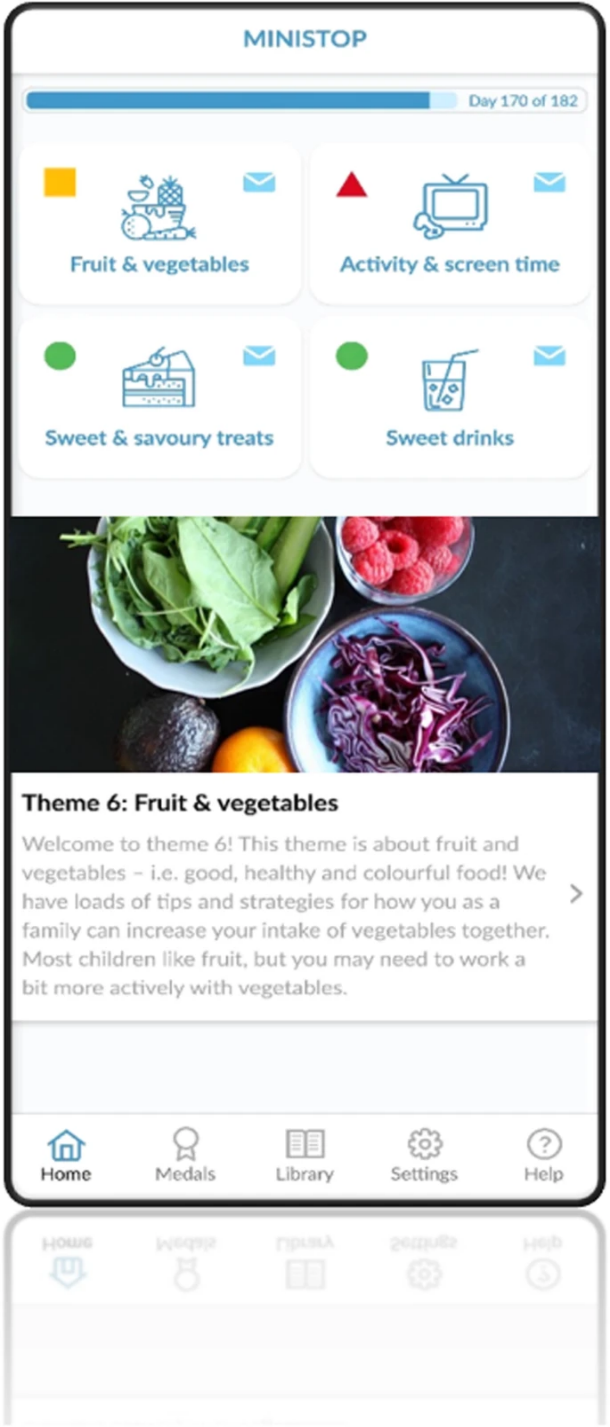


Figure 1. Screenshot of the MINISTOP 2.0 app. Adapted from Henriksson et al [1]; used under Creative Commons Attribution 4.0 License (CC BY 4.0), <https://creativecommons.org/licenses/by/4.0/>


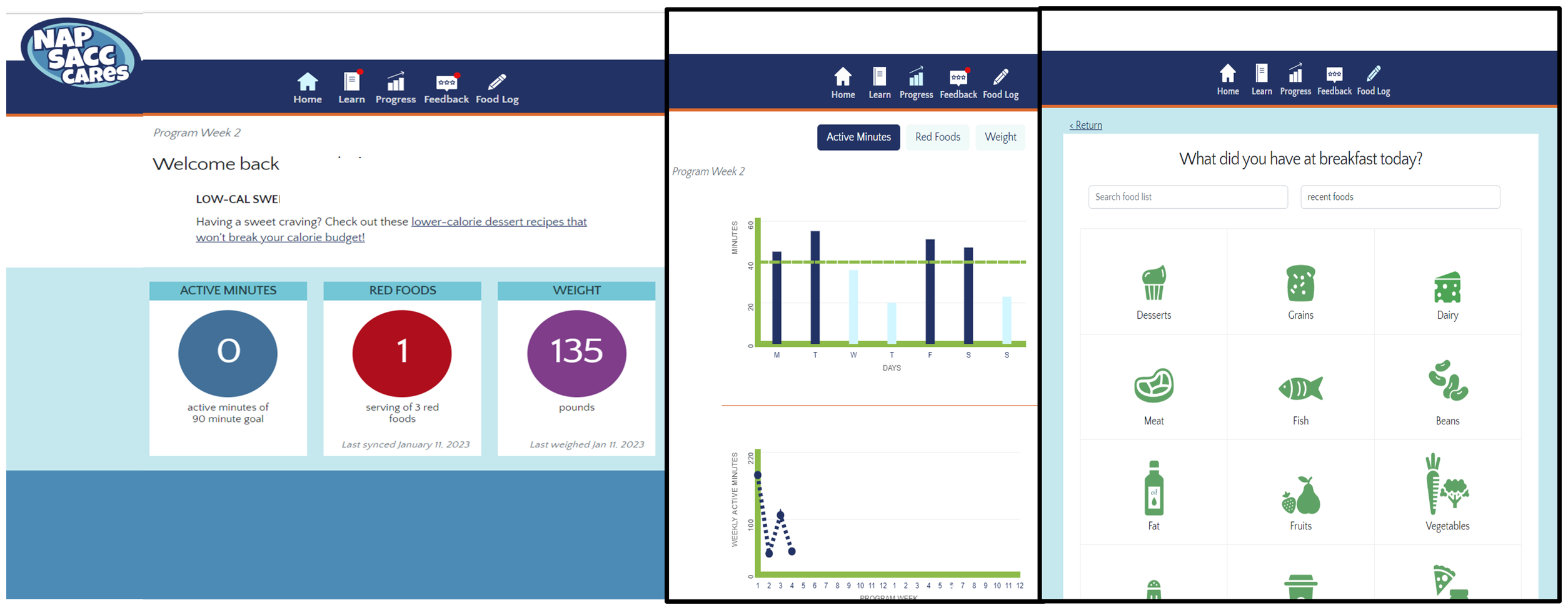


Figure 2. Screenshot of the Go NAPSACC website. The screenshot shows the feedback function in the dark blue bar and the feature to self-monitor active minutes, food, and weight. Adapted from Willis et al [2]; used under Creative Commons Attribution 4.0 License (CC BY 4.0), <https://creativecommons.org/licenses/by/4.0/>


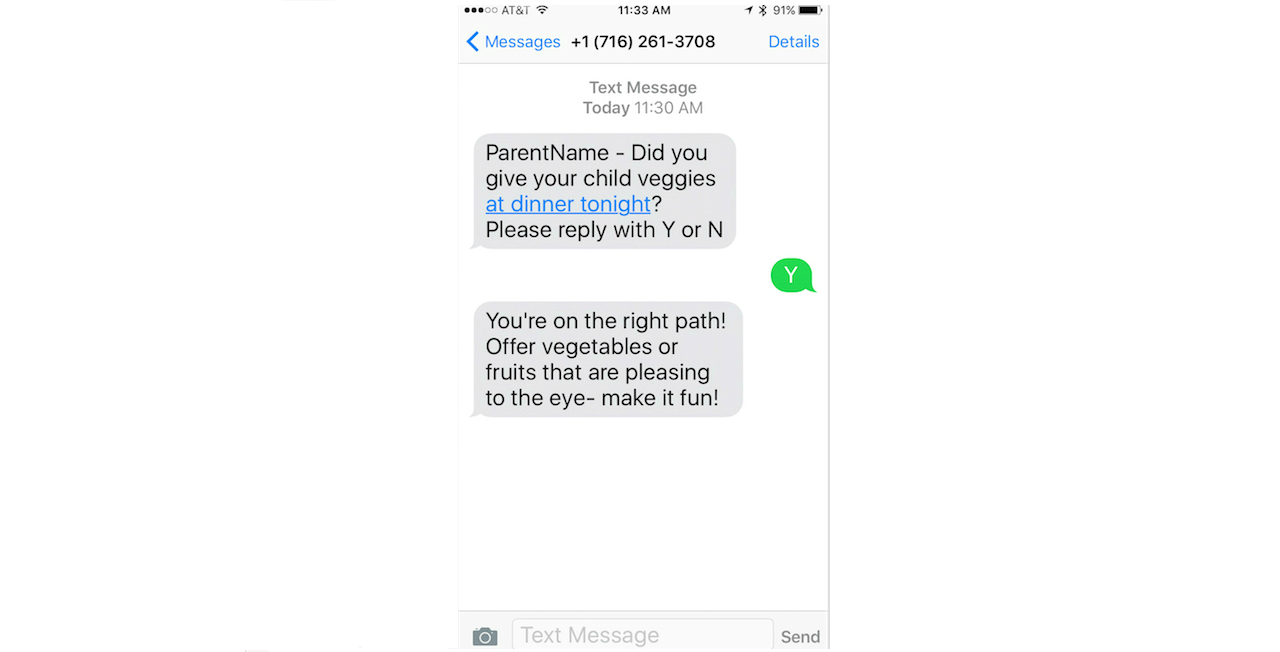


Figure 3. Screenshot of the TEXT2COPE text messages. Adapted from Militello et al [3]; used under Creative Commons Attribution 4.0 License (CC BY 4.0), <https://creativecommons.org/licenses/by/4.0/>


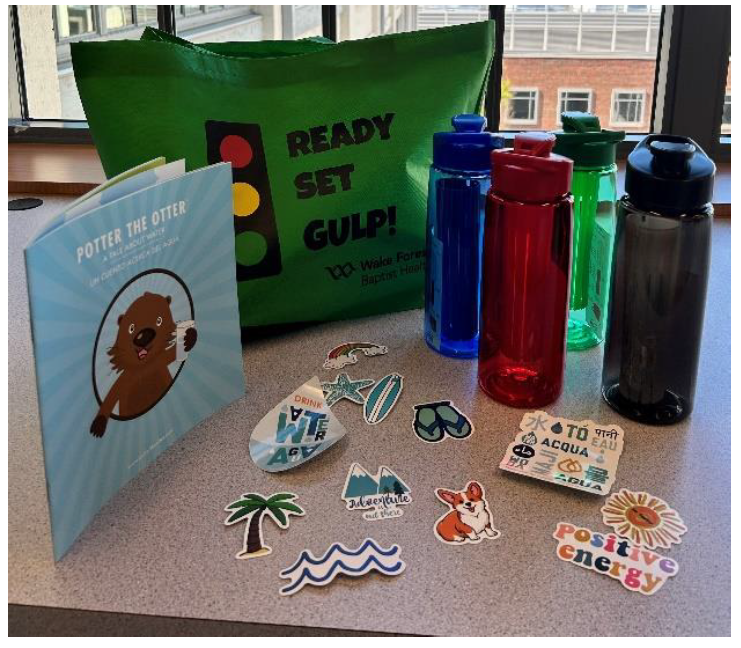


Figure 4. Photograph of the materials included in the Ready, Set, Gulp! intervention. Adapted from Lewis et al [4]; used under Creative Commons Attribution 4.0 License (CC BY 4.0), <https://creativecommons.org/licenses/by/4.0/>


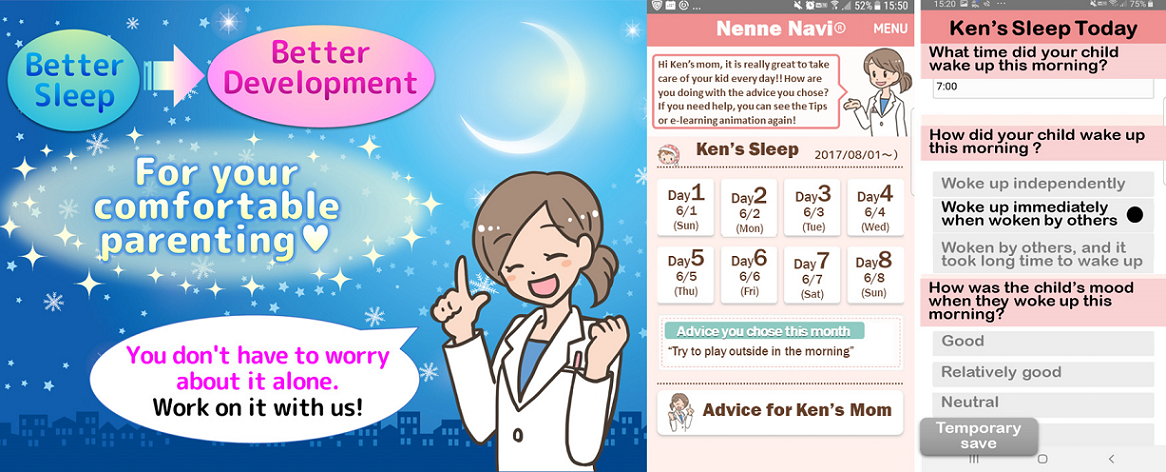


Figure 5. Screenshot of the Nenne Navi app. The screenshot shows the app giving the advice “Try to play outside in the morning”. Adapted from Yoshizaki et al [5]; used under Creative Commons Attribution 4.0 License (CC BY 4.0), <https://creativecommons.org/licenses/by/4.0/>

**References (for Multimedia Appendix 5)**

1. Henriksson H, Alexandrou C, Henriksson P, Henstrom M, Bendtsen M, Thomas K, et al. MINISTOP 2.0: a smartphone app integrated in primary child health care to promote healthy diet and physical activity behaviours and prevent obesity in preschool-aged children: protocol for a hybrid design effectiveness-implementation study. BMC Public Health. 2020 Nov 23;20(1):1756. PMID: 33228572. doi: 10.1186/s12889-020-09808-w.

2. Willis EA, Burney R, Hales D, Ilugbusi LO, Tate DF, Nezami B, et al. "My wellbeing-their wellbeing "- An eHealth intervention for managing obesity in early care and education: Protocol for the Go NAPSACC Cares cluster randomized control trial. PLoS One. 2023;18(7):e0286912. PMID: 37418363. doi: 10.1371/journal.pone.0286912.

3. Militello L, Melnyk BM, Hekler EB, Small L, Jacobson D. Automated Behavioral Text Messaging and Face-to-Face Intervention for Parents of Overweight or Obese Preschool Children: Results From a Pilot Study. JMIR Mhealth Uhealth. 2016 Mar 14;4(1):e21. PMID: 26976387. doi: 10.2196/mhealth.4398.

4. Lewis KH, Hsu FC, Block JP, Skelton JA, Schwartz MB, Krieger J, et al. A Technology-Driven, Healthcare-Based Intervention to Improve Family Beverage Choices: Results from a Pilot Randomized Trial in the United States. Nutrients. 2023 Apr 29;15(9). PMID: 37432293. doi: 10.3390/nu15092141.

5. Yoshizaki A, Mohri I, Yamamoto T, Shirota A, Okada S, Murata E, et al. An Interactive Smartphone App, Nenne Navi, for Improving Children's Sleep: Pilot Usability Study. JMIR Pediatr Parent. 2020 Dec 1;3(2):e22102. PMID: 33122163. doi: 10.2196/22102.
