# Supplementary material for: Digital and Blended Lifestyle Interventions for Preschool-Aged Children and Families With a Low Socioeconomic Position and the General Population: Scoping Review
Source: J Med Internet Res. 2026 Jun 5;28:e86596. doi: 10.2196/86596 (PMC13240985; doi:10.2196/86596)
Supplement: Multimedia Appendix 7 [file jmir-v28-e86596-s007.docx]

|  | Was true randomization used for assignment of participants to treatment groups? | Was allocation to treatment groups concealed? | Were treatment groups similar at the baseline? | Were participants blind to treatment assignment? | Were those delivering the treatment blind to treatment assignment? | Were treatment groups treated identically other than the intervention of interest? | Were outcome assessors blind to treatment assignment? | Were outcomes measured in the same way for treatment groups? | Were outcomes measured in a reliable way? | Was follow up complete and if not, were differences between groups in terms of their follow up adequately described and analysed? | Were participants analysed in the groups to which they were randomized? | Was appropriate statistical analysis used? | Was the trial design appropriate and any deviations from the standard RCT design (individual randomization, parallel groups) accounted for in the conduct and analysis of the trial? |
| --- | --- | --- | --- | --- | --- | --- | --- | --- | --- | --- | --- | --- | --- |
| Alexandrou et al [1] | ✓ | ✓ | ✓ | ✕ | N/A | ✓ | ✕ | ✓ | ? | ✓ | ✓ | ✓ | ✓ |
| Beck et al [2] | ✓ | ✓ | ✓ | ✕ | ✕ | ✓ | ? | ✓ | ? | ✓ | ✓ | ✓ | ✓ |
| Billah et al [3] | ✓ | ✓ | ✓ | ✕ | ✕ | ✓ | N/A | ✓ | ✓ | ✓ | ✓ | ✓ | ✓ |
| Blomkvist et al [4] | ✓ | ? | ✓ | ✕ | ✕ | ✓ | ✕ | ✓ | ? | ? | ✓ | ✓ | ✓ |
| Branco dos Santos Lima & Pinheiro Barbosa [5] | ✓ | ? | ✓ | ✓ | ✕ | ✓ | ✓ | ✓ | ? | ✕ | ✕ | ✕ | ✓ |
| Choonhawarakorn et al [6] | ✓ | ? | ✓ | ✕ | N/A | ✓ | ? | ✓ | ? | ✓ | ? | ✓ | ✓ |
| Downing et al [7] | ✓ | ✓ | ✓ | ✕ | N/A | ✓ | N/A | ✓ | ? | ✓ | ✓ | ✓ | ✓ |
| Gago et al [8] | ✓ | ✓ | ✕ | ✕ | ✕ | ✓ | ✓ | ✓ | ✓ | ✓ | ✓ | ✓ | ✓ |
| Hammersley et al [9] | ✓ | ✓ | ✓ | ✕ | N/A | ✓ | ✓ | ✓ | ? | ✓ | ✓ | ✓ | ✓ |
| He et al [10] | ✓ | ? | ✓ | ✓ | ? | ✓ | ✓ | ✓ | ? | ✓ | ? | ✓ | ✓ |
| Heerman et al [11] | ✓ | ✓ | ✓ | ✕ | ✕ | ✓ | ? | ✓ | ? | ✓ | ✓ | ✓ | ✓ |
| Helle et al [12] | ✓ | ✓ | ✓ | ✕ | N/A | ✓ | ? | ✓ | ? | ✓ | ✓ | ✓ | ✓ |
| Helle et al [13] | ✓ | ✓ | ✓ | ✕ | N/A | ✓ | ? | ✓ | ? | ✓ | ✓ | ✓ | ✓ |
| Ihab et al [14] | ✓ | ✓ | ✓ | ✕ | ✕ | ✓ | ? | ✓ | ? | ✓ | ✓ | ✓ | ✓ |
| Karssen et al [15] | ✓ | ✓ | ✓ | ✕ | N/A | ✓ | N/A | ✓ | ✓ | ✓ | ✓ | ✓ | ✓ |
| Lee et al [16] | ✓ | ✓ | ✓ | ✕ | N/A | ✓ | N/A | ✓ | ? | ✓ | ? | ✓ | ✓ |
| Lewis et al [17] | ✓ | ✓ | ✓ | ✕ | ✕ | ✓ | ✓ | ✓ | ? | ✓ | ✓ | ✓ | ✓ |
| Ling et al [18] | ✓ | ? | ? | ✕ | ✕ | ✓ | ? | ✓ | ? | ✓ | ✓ | ✓ | ✓ |
| Lotto et al [19] | ✓ | ✓ | ✓ | ✕ | N/A | ✓ | ✓ | ✓ | ✓ | ✓ | ✓ | ✓ | ✓ |
| Lozada-Tequeanes et al [20] | ✓ | ? | ✓ | ✕ | N/A | ✓ | ? | ✓ | ? | ✓ | ✓ | ✓ | ✓ |
| Nezami et al [21] | ✓ | ✓ | ✓ | ✕ | ✕ | ✓ | ✓ | ✓ | ✓ | ✓ | ✓ | ✓ | ✓ |
| Nyström et al [22] | ✓ | ✓ | ✓ | ✕ | N/A | ✓ | ✓ | ✓ | ✓ | ✓ | ? | ✓ | ✓ |
| Nyström et al [23] | ✓ | ✓ | ✓ | ✕ | N/A | ✓ | ✓ | ✓ | ✓ | ✓ | ? | ✓ | ✓ |
| Peden et al [24] | ✓ | ✓ | ✓ | ✕ | ✕ | ✓ | ✓ | ✓ | ✓ | ✓ | ✓ | ✓ | ✓ |
| Røed et al [25] | ✓ | ✓ | ✓ | ✕ | N/A | ✓ | N/A | ✓ | ? | ✓ | ✓ | ✓ | ✓ |
| Sandborg et al [26] | ✓ | ✓ | ✓ | ✕ | N/A | ✓ | ✕ | ✓ | ? | ✕ | ✕ | ✕ | ✓ |
| Seyyedi et al [27] | ✓ | ✓ | ✓ | ✕ | N/A | ✓ | N/A | ✓ | ? | ✓ | ✓ | ✓ | ✓ |
| Tomayko et al [28] | ✓ | ✓ | ✓ | ✕ | ✕ | ✓ | ✓ | ✓ | ? | ✓ | ? | ✓ | ✓ |
| van Grieken et al [29] | ✓ | ? | ✓ | ✕ | ✕ | ✓ | ? | ✓ | ? | ✓ | ✓ | ✓ | ✓ |
| Wu et al [30] | ✓ | ✓ | ✕ | ✕ | ✕ | ✓ | N/A | ✓ | ✓ | ✓ | ✓ | ✓ | ✓ |
| Yoshizaki et al [31] | ✕ | ✕ | ✕ | ✕ | N/A | ✓ | N/A | ✓ | ? | ✓ | ✕ | ✓ | ✕ |

**Note.** *✓ = yes; ✕ = no; ? = unsure; N/A = not applicable*

**References (for Multimedia Appendix 6)**

1. Alexandrou C, Henriksson H, Henström M, Henriksson P, Delisle Nyström C, Bendtsen M, Löf M. Effectiveness of a Smartphone App (MINISTOP 2.0) integrated in primary child health care to promote healthy diet and physical activity behaviors and prevent obesity in preschool-aged children: randomized controlled trial. Int J Behav Nutr Phys Act 2023 Feb 21;20(1):22. doi: 10.1186/s12966-023-01405-5

2. Beck AL, Mora R, Joseph G, Perrin E, Cabana M, Schickedanz A, Fernandez A. A Multimethod Evaluation of the Futuros Fuertes Intervention to Promote Healthy Feeding, Screen Time, and Sleep Practices. Academic Pediatrics 2023 Sept;23(7):1351–1360. doi: 10.1016/j.acap.2023.05.006

3. Billah SM, Ferdous TE, Kelly P, Raynes‐Greenow C, Siddique AB, Choudhury N, Ahmed T, Gillespie S, Hoddinott J, Menon P, Dibley MJ, Arifeen SE. Effect of nutrition counselling with a digital job aid on child dietary diversity: Analysis of secondary outcomes from a cluster randomised controlled trial in rural Bangladesh. Maternal & Child Nutrition 2022 Jan;18(1):e13267. doi: 10.1111/mcn.13267

4. Blomkvist EAM, Wills AK, Helland SH, Hillesund ER, Øverby NC. Effectiveness of a kindergarten-based intervention to increase vegetable intake and reduce food neophobia amongst 1-year-old children: a cluster randomised controlled trial. Food & Nutrition Research 2021 Oct 8;65. doi: 10.29219/fnr.v65.7679

5. Branco Dos Santos Lima R, Barbosa LP. Self-efficacy-based web and mobile intervention to improve infant sleep: Randomized trial with Brazilian mothers. Sleep Medicine 2025 Oct;134:106718. doi: 10.1016/j.sleep.2025.106718

6. Choonhawarakorn K, Kasemkhun P, Leelataweewud P. Effectiveness of a message service on child oral health practice via a social media application: A randomized controlled trial. Int J Paed Dentistry 2025 Mar;35(2):446–455. doi: 10.1111/ipd.13256

7. Downing KL, Salmon J, Hinkley T, Hnatiuk JA, Hesketh KD. Feasibility and Efficacy of a Parent-Focused, Text Message–Delivered Intervention to Reduce Sedentary Behavior in 2- to 4-Year-Old Children (Mini Movers): Pilot Randomized Controlled Trial. JMIR Mhealth Uhealth 2018 Feb 9;6(2):e39. doi: 10.2196/mhealth.8573

8. Gago C, Aftosmes-Tobio A, Beckerman-Hsu JP, Oddleifson C, Garcia EA, Lansburg K, Figueroa R, Yu X, Kitos N, Torrico M, Leonard J, Jurkowski JK, Mattei J, Kenney EL, Haneuse S, Davison KK. Evaluation of a cluster-randomized controlled trial: Communities for Healthy Living, family-centered obesity prevention program for Head Start parents and children. Int J Behav Nutr Phys Act 2023 Jan 11;20(1):4. doi: 10.1186/s12966-022-01400-2

9. Hammersley ML, Okely AD, Batterham MJ, Jones RA. An Internet-Based Childhood Obesity Prevention Program (Time2bHealthy) for Parents of Preschool-Aged Children: Randomized Controlled Trial. J Med Internet Res 2019 Feb 8;21(2):e11964. doi: 10.2196/11964

10. He Q, Ha ASC, Zheng B, Okely AD. Feasibility and potential efficacy of a family‐based intervention on promoting physical activity levels and fundamental movement skills in preschoolers: A cluster randomised controlled trial. Applied Psych Health &amp; Well 2024 Aug;16(3):1266–1288. doi: 10.1111/aphw.12527

11. Heerman WJ, Rothman RL, Sanders LM, Schildcrout JS, Flower KB, Delamater AM, Kay MC, Wood CT, Gross RS, Bian A, Adams LE, Sommer EC, Yin HS, Perrin EM, Greenlight Investigators, De La Barrera B, Bility M, Cruz Jimenez Smith M, Cruzatte EF, Guevara G, Howard JB, Lampkin J, Orr CJ, Pilotos McBride J, Quintana Forster L, Ramirez KS, Rodriguez J, Schilling S, Shepard WE, Soto A, Velazquez JJ, Wallace S. A Digital Health Behavior Intervention to Prevent Childhood Obesity: The Greenlight Plus Randomized Clinical Trial. JAMA 2024 Dec 24;332(24):2068. doi: 10.1001/jama.2024.22362

12. Helle C, Hillesund ER, Wills AK, Øverby NC. Evaluation of an eHealth intervention aiming to promote healthy food habits from infancy -the Norwegian randomized controlled trial Early Food for Future Health. Int J Behav Nutr Phys Act 2019 Dec;16(1):1. doi: 10.1186/s12966-018-0763-4

13. Helle C, Hillesund ER, Wills AK, Øverby NC. Examining the effects of an eHealth intervention from infant age 6 to 12 months on child eating behaviors and maternal feeding practices one year after cessation: The Norwegian randomized controlled trial Early Food for Future Health. Simeoni U, editor. PLoS ONE 2019 Aug 23;14(8):e0220437. doi: 10.1371/journal.pone.0220437

14. Ihab M, El-Sherif Y, Yassin R, Nabil N, Quritum M, Balbaa N, Tantawi ME. Optimizing mHealth Interventions for Children’s Oral Hygiene: A Factorial Trial. J Dent Res 2025 Feb;104(2):155–163. doi: 10.1177/00220345241291985

15. Karssen LT, Larsen JK, Burk WJ, Kremers SPJ, Hermans RCJ, Ruiter ELM, Vink JM, De Weerth C. Process and effect evaluation of the app-based parenting program Samen Happie! on infant zBMI: A randomized controlled trial. Front Public Health 2022 Dec 23;10:1012431. doi: 10.3389/fpubh.2022.1012431

16. Lee H, Oldewage-Theron W, Dawson J. Effects of a Theory-Based, Multicomponent eHealth Intervention for Obesity Prevention in Young Children from Low-Income Families: A Pilot Randomized Controlled Study. Nutrients 2023 May 13;15(10):2296. doi: 10.3390/nu15102296

17. Lewis KH, Hsu F-C, Block JP, Skelton JA, Schwartz MB, Krieger J, Hindel LR, Ospino Sanchez B, Zoellner J. A Technology-Driven, Healthcare-Based Intervention to Improve Family Beverage Choices: Results from a Pilot Randomized Trial in the United States. Nutrients 2023 Apr 29;15(9):2141. doi: 10.3390/nu15092141

18. Ling J, Suriyawong W, Robbins LB, Zhang N, Kerver JM. FirstStep2Health : A cluster randomised trial to promote healthy behaviours and prevent obesity amongst low‐income preschoolers. Pediatric Obesity 2024 July;19(7):e13122. doi: 10.1111/ijpo.13122

19. Lotto M, Strieder AP, Ayala Aguirre PE, Oliveira TM, Andrade Moreira Machado MA, Rios D, Cruvinel T. Parental-oriented educational mobile messages to aid in the control of early childhood caries in low socioeconomic children: A randomized controlled trial. Journal of Dentistry 2020 Oct;101:103456. doi: 10.1016/j.jdent.2020.103456

20. Lozada-Tequeanes AL, Théodore FL, Kim-Herrera E, García-Guerra A, Quezada-Sánchez AD, Alvarado-Casas R, Bonvecchio A. Effectiveness and Implementation of a Text Messaging mHealth Intervention to Prevent Childhood Obesity in Mexico in the COVID-19 Context: Mixed Methods Study. JMIR Mhealth Uhealth 2024 Apr 9;12:e55509. doi: 10.2196/55509

21. Nezami BT, Ward DS, Lytle LA, Ennett ST, Tate DF. A mHealth randomized controlled trial to reduce sugar‐sweetened beverage intake in preschool‐aged children. Pediatric Obesity 2018 Nov;13(11):668–676. doi: 10.1111/ijpo.12258

22. Nyström CD, Sandin S, Henriksson P, Henriksson H, Trolle-Lagerros Y, Larsson C, Maddison R, Ortega FB, Pomeroy J, Ruiz JR, Silfvernagel K, Timpka T, Löf M. Mobile-based intervention intended to stop obesity in preschool-aged children: the MINISTOP randomized controlled trial ,. The American Journal of Clinical Nutrition 2017 June;105(6):1327–1335. doi: 10.3945/ajcn.116.150995

23. Nyström, CD, Sandin S, Henriksson P, Henriksson H, Maddison R, Löf M. A 12-month follow-up of a mobile-based (mHealth) obesity prevention intervention in pre-school children: the MINISTOP randomized controlled trial. BMC Public Health 2018 Dec;18(1):658. doi: 10.1186/s12889-018-5569-4

24. Peden M, Eady M, Okely A, Patterson K, Batterham M, Jones R. A blended professional learning intervention for early childhood educators to target the promotion of physical activity and healthy eating: the HOPPEL cluster randomized stepped-wedge trial. BMC Public Health 2022 Dec;22(1):1353. doi: 10.1186/s12889-022-13542-w

25. Røed M, Medin AC, Vik FN, Hillesund ER, Van Lippevelde W, Campbell K, Øverby NC. Effect of a Parent-Focused eHealth Intervention on Children’s Fruit, Vegetable, and Discretionary Food Intake (Food4toddlers): Randomized Controlled Trial. J Med Internet Res 2021 Feb 16;23(2):e18311. doi: 10.2196/18311

26. Sandborg J, Downing KL, Orellana L, Taylor RW, Barnett LM, Carson V, Hesketh KD. Six-month intervention effect of a digital movement behavior intervention on parent- and child intermediary outcomes—results from the Let’s Grow randomized controlled trial. Int J Behav Nutr Phys Act 2025 June 16;22(1):78. doi: 10.1186/s12966-025-01764-1

27. Seyyedi N, Rahimi B, Eslamlou HRF, Afshar HL, Spreco A, Timpka T. Smartphone-Based Maternal Education for the Complementary Feeding of Undernourished Children Under 3 Years of Age in Food-Secure Communities: Randomised Controlled Trial in Urmia, Iran. Nutrients 2020 Feb 24;12(2):587. doi: 10.3390/nu12020587

28. Tomayko EJ, Prince RJ, Cronin KA, Kim K, Parker T, Adams AK. The Healthy Children, Strong Families 2 (HCSF2) Randomized Controlled Trial Improved Healthy Behaviors in American Indian Families with Young Children. Current Developments in Nutrition 2019 Aug;3:53–62. doi: 10.1093/cdn/nzy087

29. Van Grieken A, Vlasblom E, Wang L, Beltman M, Boere-Boonekamp MM, L’Hoir MP, Raat H. Personalized Web-Based Advice in Combination With Well-Child Visits to Prevent Overweight in Young Children: Cluster Randomized Controlled Trial. J Med Internet Res 2017 July 27;19(7):e268. doi: 10.2196/jmir.7115

30. Wu Q, Wang X, Zhang J, Zhang Y, Van Velthoven MH. The effectiveness of a WeChat-based self-assessment with a tailored feedback report on improving complementary feeding and movement behaviour of children aged 6–20 months in rural China: a cluster randomized controlled trial. The Lancet Regional Health - Western Pacific 2023 Aug;37:100796. doi: 10.1016/j.lanwpc.2023.100796

31. Yoshizaki A, Murata E, Yamamoto T, Fujisawa TX, Hanaie R, Hirata I, Matsumoto S, Mohri I, Taniike M. Improving Children’s Sleep Habits Using an Interactive Smartphone App: Community-Based Intervention Study. JMIR Mhealth Uhealth 2023 Feb 10;11:e40836. doi: 10.2196/40836
