# Supplementary material for: Digital and Blended Lifestyle Interventions for Preschool-Aged Children and Families With a Low Socioeconomic Position and the General Population: Scoping Review
Source: J Med Internet Res. 2026 Jun 5;28:e86596. doi: 10.2196/86596 (PMC13240985; doi:10.2196/86596)
Supplement: Multimedia Appendix 8 [file jmir-v28-e86596-s008.docx]

|  | Is it clear in the study what is the “cause” and what is the “effect” (i.e. there is no confusion about which variable comes first)? | Was there a control group? | Were participants included in any comparisons similar? | Were the participants included in any comparisons receiving similar treatment/care, other than the exposure or intervention of interest? | Were there multiple measurements of the outcome, both pre and post the intervention/exposure? | Were the outcomes of participants included in any comparisons measured in the same way? | Were outcomes measured in a reliable way? | Was follow-up complete and if not, were differences between groups in terms of their follow-up adequately described and analyzed? | Was appropriate statistical analysis used? |
| --- | --- | --- | --- | --- | --- | --- | --- | --- | --- |
| Downs et al [1] | ✓ | ✓ | ✓ | ✓ | ✓ | ✓ | ? | ✓ | ✓ |
| Ghofranipour et al [2] | ✓ | ✕ | ✓ | ✓ | ✓ | ✓ | ? | ✓ | ✕ |
| Jiying et al [3] | ✓ | ✓ | ✕ | ✓ | ✓ | ✓ | ? | ✕ | ✓ |
| Militello et al [4] | ✓ | ✕ | ✓ | ✓ | ✓ | ✓ | ? | ✓ | ✓ |
| Zhang et al [5] | ✓ | ✕ | ✓ | ✓ | ? | ✓ | ? | ✓ | ✓ |

**Note.** *✓ = yes; ✕ = no; ? = unsure; N/A = not applicable*

**References (for Multimedia Appendix 7)**

1. Downs SM, Sackey J, Kalaj J, Smith S, Fanzo J. An mHealth voice messaging intervention to improve infant and young child feeding practices in Senegal. Maternal & Child Nutrition 2019 Oct;15(4):e12825. doi: 10.1111/mcn.12825

2. Ghofranipour F, Hamzavi Zarghani N, Mohammadi E, Mehrizi AAH, Tavousi M, De Craemer M, Cardon G. An internet-based educational intervention for mothers targeting preschoolers’ weight management promotion (PWMP): a pilot study. BMC Public Health 2022 Nov 29;22(1):2220. doi: 10.1186/s12889-022-14543-5

3. Jiying L, Sisi C, Nanhua Z, Lorraine B. R, Jean M. K. Happy Family, Healthy Kids: A Healthy Eating and Stress Management Program in Low-Income Parent–Preschooler Dyads. Nursing Research 2024 Jan;73(1):3–15. doi: 10.1097/NNR.0000000000000697

4. Militello L, Melnyk BM, Hekler EB, Small L, Jacobson D. Automated Behavioral Text Messaging and Face-to-Face Intervention for Parents of Overweight or Obese Preschool Children: Results From a Pilot Study. JMIR mHealth uHealth 2016 Mar 14;4(1):e21. doi: 10.2196/mhealth.4398

5. Zhang Q, Panichelli J, Hall LA. Assessment of Cooking Matters Facebook Platform to Promote Healthy Eating Behaviors among Low-Income Caregivers of Young Children in the United States: A Pilot Study. Nutrients 2021 Aug 4;13(8):2694. doi: 10.3390/nu13082694
